# Additive effect of tDCS and neuromotor recruitment on functional recovery in chronic paraplegia: A randomized controlled trial

**DOI:** 10.1371/journal.pone.0352320

**Published:** 2026-06-23

**Authors:** Ahmad Rifai Sarraj, Jihan Allaw, Eliane Rached, Joy Khayat, Hassan Karaki, Ahmad Diab, Antonio Pinti

**Affiliations:** 1 Faculty of Public Health, Laboratory of Motor System, Handicap and Rehabilitation (MOHAR), Lebanese University, Beirut, Lebanon; 2 Laboratoire de Recherche Sociétés et Humanité (LARSH DeVisu), Université Polytechnique Hauts-De-France, Valenciennes, France; 3 Biomedical Engineering Department, Signal Processing Research Group, Lebanese International University, Tripoli, Lebanon; 4 Biomedical Engineering Department, Signal Processing Research Group, The International University IU, Beirut, Lebanon; TIU: Tishk International University, IRAQ

## Abstract

Functional recovery in chronic spinal cord injury (SCI) is traditionally viewed as limited, particularly after the spontaneous recovery window has closed. This single-blind randomized controlled trial investigated whether adding anodal Transcranial Direct Current Stimulation (tDCS) to the Neuromotor Recruitment Method (NEUROM)—a protocol combining motor imagery with intensive peripheral sensory stimulation—provides a additive benefit for sensorimotor recovery. Fifty participants with chronic paraplegia (mean time since injury: 15.4 ± 3.2 months; ASIA Impairment Scale A, B, or C) were recruited and randomized into three arms: Reference (standard care, n = 10), NEUROM (n = 20), or NEUROM+tDCS (n = 20). The intervention was administered over 10 days. Outcomes included the Lower Extremity Motor Score (LEMS), Light Touch and Pin Prick sensory scores, and the Assessment of Movement Attempt (AMA). The Reference group showed no significant recovery. Both active groups (NEUROM and NEUROM+tDCS) achieved substantial and statistically identical improvements in sensory function compared to the Reference group (*p* < 0.001), suggesting that peripheral recruitment alone is sufficient for afferent restoration. However, a significant dissociation was observed in motor function: the NEUROM+tDCS group demonstrated superior LEMS recovery (Mean change: 18 points) compared to the NEUROM group (14 points; *p* = 0.01) and reported significantly higher volitional drive (*p* < 0.001). These findings indicate a clear dissociation between sensory and motor plasticity in chronic SCI; while peripheral somatosensory recruitment drives afferent sensory restoration, the addition of central stimulation via tDCS is critical for maximizing efferent motor output. This suggests that restoring motor function in chronic paraplegia requires a “top-down” cortical prime to complement “bottom-up” peripheral signaling. Trial Registration: ClinicalTrials.gov NCT04790149.

## Introduction

Spinal cord injury (SCI) is a debilitating condition that results in the loss of motor and sensory functions. Historically, the spinal cord has been described as a protected bundle of nerves connecting the brain to the body, mediating motor, sensory, and some autonomic functions below the neck (for a historical review, see [1], also [2]). Traumatic Spinal Cord Injury (TSCI) is a catastrophic and unexpected event that can occur along the spinal column (cervical, thoracic, and lumbar). The global incidence rate (2007) is estimated at 23 TSCI cases per million, totaling approximately 179,312 cases annually [3]. TSCI often results in life-threatening conditions, including varying degrees of motor paralysis, sensory loss, and impairment of bowel, bladder, sexual, and other physiological functions. Recent studies highlight that TSCI can lead to severe and potentially life-threatening syndromes such as autonomic dysreflexia [4].

Reorganization of the central nervous system (CNS) after injury has been demonstrated in both animals and humans, improving our understanding of the repair processes that can occur through various mechanisms [5]. Spontaneous functional recovery after SCI may depend on substantial reorganization of propriospinal circuits and the amount of spared axons that can contribute to functional gains over time [6]. Additionally, surviving fibers may produce adaptive rewiring of spared interneurons, significantly impacting post-SCI plasticity and enabling patients to regain their previous motor and functional abilities [6].

Operant conditioning protocols in animals and humans, including activation of the corticospinal tract, can produce multiple structural changes and plasticity in the spinal cord. These changes include modifications in motor neuron firing thresholds, axonal conduction velocity, and synaptic terminals on motor neurons. This activity-dependent plasticity, driven by input from the periphery and/or the brain, plays a crucial role in the acquisition and maintenance of motor skills after SCI [7].

Neurorehabilitation has been demonstrated to be effective in inducing and guiding histological and/or functional recovery in SCI patients [8]. Clinical trials recommend replacing conventional and compensatory rehabilitative strategies, which often involve braces, assistive devices, and wheelchairs, with restorative strategies. These restorative therapies aim to achieve activation of the neurologically injured level through function-specific motor tasks and intensive exercise training, optimizing functional, metabolic, and neurological status in SCI patients [9–12].

Functional magnetic resonance imaging (fMRI) studies have found strong associations between motor task-related fMRI activation and the degree of motor function post-SCI. For instance, increased fMRI activation within sensorimotor and premotor networks has been correlated with improved locomotor function and motor skill acquisition after SCI [13]. Similarly, the extent of activation in the primary motor cortex (M1) is often linked to the degree of motor recovery, suggesting that motor imagery, action observation, and other cognitive stimuli combined with functional and restorative exercises can effectively engage larger cortical networks, leading to better recovery outcomes [14].

Following SCI, the spinal cord is deprived of descending inputs from the brain. However, sensory inputs may still persist, and peripheral sensory inputs can target adaptive changes in spared axons within below-level neuronal networks of the spinal cord. For example, muscle stretching can generate specific peripheral sensory input in muscle receptors, potentially provoking central sensitization and facilitating adaptive plasticity [15].

These findings highlight the importance of integrating motor imagery and peripheral sensory inputs into rehabilitation protocols to promote neuroplasticity and functional recovery in SCI patients. The combination of these techniques can enhance the activation of cortical and spinal networks, leading to significant improvements in motor and sensory functions.

In this study, we propose an experimental rehabilitative protocol for TSCI patients based on the histological and/or functional reorganization model of the spinal cord after injury, as well as activity-dependent plasticity and motor imagery. This method may enhance sparing-induced plasticity and facilitate learned-reflex modulation within interneuron networks. Furthermore, non-invasive brain stimulation, specifically Transcranial Direct Current Stimulation (tDCS), has been shown to modulate cortical excitability and prime the motor cortex for learning [16,17]. We hypothesized that “priming” the cortex with tDCS prior to the NEUROM protocol would create an additive effect, enhancing functional recovery more than either modality alone.

## Materials and methods

### Study design and participants

This study utilized a randomized, controlled, single-blind experimental design conducted over a period of 3 weeks. The study protocol was registered at ClinicalTrials.gov (Identifier: NCT04790149). We acknowledge that the administrative registration of this trial was completed after the initial ethical approval was granted. This delay was directly caused by the onset of the COVID-19 pandemic, which necessitated a strict, institutional freeze on patient recruitment across our multicenter clinical sites for the safety of this vulnerable population. Consequently, the practical start of the study and the initiation of active patient enrollment were delayed until December 1, 2022. We confirm that despite this administrative disruption, the study protocol remained strictly aligned with the original ethical approval. Absolutely no modifications or changes were made to the study design, interventions, or procedures after registration. The authors confirm that all ongoing and related trials for this drug/intervention are registered.

**Fig 1:**
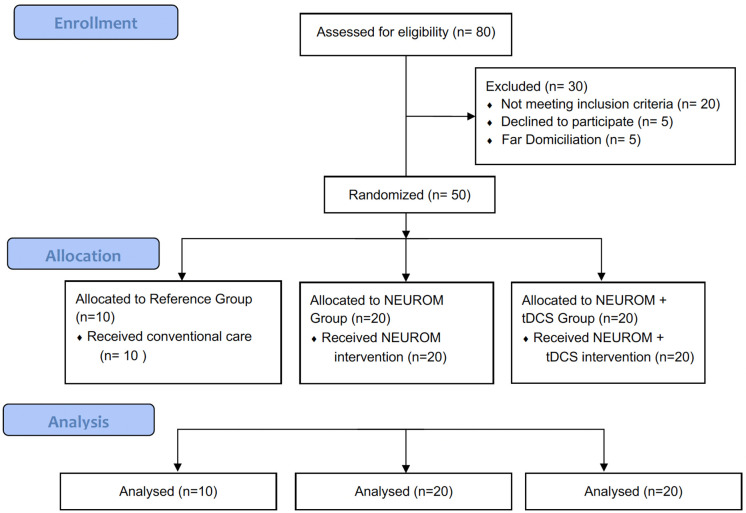
CONSORT flow diagram displaying the progress of all participants through the trial, including enrollment, intervention allocation, follow-up, and data analysis. Analysis was performed on an intention-to-treat basis with no participants lost to follow-up.

As a multicenter study, the protocol underwent rigorous prospective review and was approved by the respective Institutional Review Boards, including the Ethics Committee at the Centre Azm for Research in Biotechnology (Lebanese University, CE-EDST-10–2019) and the medical administration at Rahma Hospital for Rehabilitation. A single, comprehensive original approval document covered the entire study period, verifying that formal ethical oversight was established well before the trial’s practical inception and maintained throughout. The flow of all participants through the screening, enrollment, randomization, and follow-up phases is illustrated in Fig 1.

Participants were screened by a specialized physician and excluded if they had major psychiatric diagnoses, cognitive impairments limiting instruction following, contraindications to tDCS, or were using medications known to inhibit tDCS effects (e.g., anti-epileptics, dopaminergics).

### Blinding

Due to the distinct and active nature of the therapeutic protocols (e.g., active motor imagery and peripheral sensory stimulation versus conventional physical therapy), it was not feasible to blind the participants or the treating therapists to the group allocation. Therefore, the study was designed as a single-blind trial with strictly blinded outcome assessment. The Outcome Assessors—specialized physical therapists responsible for performing all baseline and post-intervention evaluations (ISNCSCI/ASIA and AMA)—were completely blinded to the participants’ group allocations and were not involved in delivering any treatment interventions. To maintain the integrity of this blinding, participants were explicitly instructed not to discuss their assigned treatment protocols, daily activities, or the use of neurostimulation equipment with the assessors. Furthermore, the data analysts performed all statistical analyses on coded datasets without knowledge of the specific intervention received by each group.

### Randomization and allocation concealment

The random allocation sequence was generated using a web-based computer random number generator (www.random.org) to ensure a truly random and unbiased assignment. The generation process was performed by an independent investigator who was not involved in participant recruitment, treatment, or clinical assessment. Participants were allocated in a 1:2:2 ratio to the Reference (*n* = 10), NEUROM (*n* = 20), or NEUROM+tDCS (*n* = 20) groups, respectively. Allocation concealment was maintained by revealing the group assignment to the treating therapists only after the participant had completed baseline assessments and signed the informed consent.

### Experimental procedure

Following baseline assessment (Day 1), participants were randomized into one of three distinct intervention arms: the Reference Group (standardized conventional care), the NEUROM Group (motor imagery combined with peripheral sensory stimulation), and the NEUROM+tDCS Group (cortical priming followed by the NEUROM protocol).

To ensure strict reproducibility, treatment fidelity, and standardized application across all therapists, the complete step-by-step methodology for these interventions has been deposited in protocols.io. This includes the precise sequencing of the sensory-motor recruitment phases, as well as the evidence-based rationale and neurophysiological justification for the selected tDCS parameters (1.0 mA, bilateral anodal stimulation over M1). The full, open-access protocol is available at: https://dx.doi.org/10.17504/protocols.io.5qpvoe6bzl4o/v1.

1. **Reference Group (Control, n=10) (3 weeks).**

Participants in this group received a standardized conventional rehabilitation protocol that was implemented to account for spontaneous recovery and the influence of routine interaction. Each session comprised a structured regimen beginning with 20 minutes of both passive and active mobilization exercises aimed at maintaining joint flexibility and muscle activity. This was followed by 15 minutes of standing using a passive standing frame, providing postural support and weight-bearing stimulation without active effort from the patient. The session concluded with 10 minutes focused on sitting posture training to enhance trunk control and stability. Notably, participants in this protocol did not receive any electrical stimulation or specific motor imagery training, ensuring that any observed improvements could be attributed to the standardized rehabilitation activities alone.

1. **NEUROM Group (n=20)**

Participants in the NEUROM group (n = 20) underwent the Neuromotor Recruitment Method, a protocol designed to integrate central motor planning with peripheral sensory feedback through a structured three-phase approach.

**Phase 1**: Motor Imagery Training (One week- 6 days)

**Fig 2:**
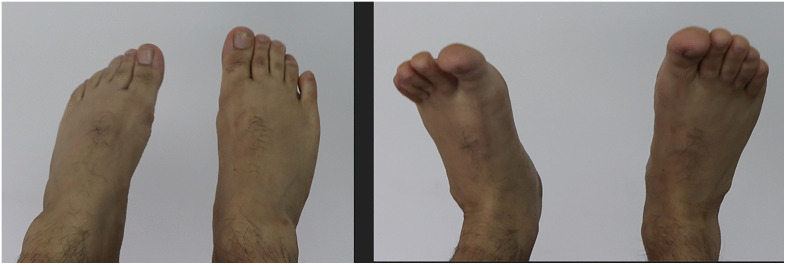
Screenshots from the video showed to participants in the AO phase.

These daily sessions involve static stretching of the lower limbs, specifically the hamstrings and triceps muscles, for 15 minutes. Each stretching bout lasts for 2 minutes without rest. Following the stretching exercises, patients undergo motor imagery training, which consists of three different modalities described as follows:

a **Action observation**: Patients watch a short video demonstrating repetitive plantar and dorsal flexion movements (Fig 2). The video lasts for 1 minute, with auditory feedback provided at a tempo of 20 beats per minute (bpm).
b **Visual imagery in the third-person perspective**: Patients are instructed to vividly create a mental image of the observed video (dorsiflexion-plantar flexion) for one minute. This is done in the same position and in a darkened treatment room. The start and end of the imagined movement are guided by the same auditory cues of 20 bpm.
c **Kinesthetic imagery**: The physical therapist guides the patient to imagine performing the same foot movement without physically executing it. Patients are instructed to close their eyes during this phase and focus on feeling the movement.

These three phases of motor imagery training are repeated in 20 trials with 2 sets per session.

To minimize expectation effects, stimuli are presented in a pseudo-randomized order between phase 2 and 3 of the motor imagery training for each trial. Patients are instructed to maintain a forward gaze throughout the entire experiment. Verbal instructions are delivered binaurally through earphones using Presentation software (Neurobehavioral Systems® [18], version 25). All auditory materials and verbal instructions are recorded and processed using Audacity (version 3.0.3). The instructions are normalized to ensure equal sound levels.

**Phase 2**: NEUROM (2 weeks – 4 sessions/week)

In each session of this phase, the motor imagery (MI) training described in Phase 1 is reiterated at the outset and prior to commencing the NEUROM protocol. The MI training consists of the patients performing 2 sets of imagery, with 20 repetitions per set. Additionally, a global stretching of the muscles in the lower limbs is conducted to prepare for the introduction of the MI training in the NEUROM protocol.

To begin the NEUROM protocol, the patient is given the following instructions while keeping their eyes closed: “while eyes closed, try to remember the movements that were just demonstrated and experienced during the MI training, try to visualize the movements and reestablish a connection with both feet.” During this imagery exercise, the therapist incorporates a series of superficial sensory stimuli, including tactile vibrations, as well as global and rapid stretching of the lower limbs. The trial concludes by prompting the patients to move their ankles in any direction, making use of their maximum strength. This process of sensory sensitization and motor recruitment is repeated approximately 10 times, with variations in limb position, while additionally employing sensitization and facilitation techniques such as limb abduction, hamstring stretching, and dorsi/plantar flexor stretching of the ankles.

**Phase 3**: Functional Rehabilitation (Standing between parallel bars, waking with technical aids if possible).

1. **NEUROM + tDCS Group (n=20)**

The NEUROM + tDCS group (n = 20) followed the same Neuromotor Recruitment Method protocol described previously, with the addition of transcranial direct current stimulation (tDCS) administered prior to the physical therapy exercises to prime the motor cortex. Bilateral anodal tDCS was delivered using a StarStim multi-focal device (Neuroelectrics), with saline-soaked sponge electrodes measuring 35 cm² placed over the primary motor cortex (M1) of each hemisphere at locations C3 and C4 according to the 10–20 EEG system. The stimulation involved a current of 1.0 mA applied for 15 minutes on each side, totaling 30 minutes of stimulation, with a 30-second ramp-up and ramp-down period. The cathode electrodes were positioned over the contralateral supraorbital area. After the tDCS session, participants rested for 15 minutes before immediately beginning the NEUROM protocol, thereby integrating cortical priming with the established motor imagery, sensory-motor recruitment, and functional application phases.

Briefly, the tDCS parameters for the combined group were selected based on established neurophysiological evidence. A 1.0 mA intensity was chosen over higher doses to provide a stable increase in corticospinal excitability while avoiding unpredictable, calcium-dependent plasticity shifts—such as cathodal stimulation paradoxically shifting from inhibition to facilitation at higher intensities [19,20]. Furthermore, a bilateral anodal montage (35 cm² electrodes over C3 and C4) was utilized to address the bilateral motor deficits inherent to paraplegia, simultaneously priming the leg motor representations of both hemispheres prior to peripheral neuromotor recruitment [21].

### Safety and adverse event monitoring

Although tDCS is widely recognized as a safe, non-invasive neuromodulation technique with no reports of serious adverse effects at intensities of 1–2 mA [22], mild and transient side effects—such as headache, cutaneous sensation (itching/tingling), fatigue, and skin redness—have been documented in the literature [23,24]. To ensure participant safety, a rigorous monitoring protocol was implemented throughout the study. First, all participants underwent a comprehensive pre-screening by a neurologist to rule out contraindications (e.g., history of seizures, metal implants). Second, all stimulation sessions were directly supervised by a senior neurorehabilitation specialist to monitor for immediate adverse reactions. Third, a structured safety questionnaire was administered immediately before and after each tDCS session to quantify any emerging symptoms (e.g., headache, scalp irritation, nausea, or acute mood changes). A pre-defined “stopping rule” was established wherein any reported symptom severity of 5/10 would result in immediate cessation of the session and withdrawal from the study; however, no severe adverse events or study withdrawals related to safety occurred.

### Outcome measures

The primary outcome measures for this trial were the objective neurological assessments of motor and sensory recovery, specifically the Lower Extremity Motor Score (LEMS) and the ISNCSCI Light Touch and Pin Prick scores. The secondary outcome measure was the subjective evaluation of volitional drive using the Assessment of Movement Attempt (AMA) Scale.

### International Standards for Neurological Classification of Spinal Cord Injury (ISNCSCI)/ ASIA Protocols

The ISNCSCI provides the standardized method for neurological assessment after spinal cord injury, covering detailed sensory and motor scoring. Sensory evaluation tests 28 dermatomes bilaterally for light touch and pin prick on a 0–2 scale, summing to maximum scores of 112 each. Motor assessment focuses on key muscle groups with strength graded on the 0–5 Medical Research Council (MRC) scale. The guidelines also include classification into ASIA Impairment Scale (AIS) grades A through D based on sensory and motor completeness/incompleteness. This protocol is detailed in the official ISNCSCI booklet and foundational papers by the American Spinal Injury Association (ASIA) [25,26].

### Lower Extremity Motor Score (LEMS)

The LEMS is a reliable metric for quantifying motor function in the lower limbs after spinal cord injury. It assesses five key muscle groups per leg in line with neurological myotomes (L2-S1), graded on the MRC 6-point scale. The total possible score is 50, with higher scores indicating better motor recovery. LEMS correlates well with ambulatory capabilities and has been widely used in clinical trials and outcome prediction studies [27–29].

### Assessment of Movement Attempt (AMA) Scale

The AMA scale evaluates volitional motor drive by asking patients to attempt movement of a paralyzed limb, with self-reported ratings for intensity and frequency of movement attempts. This subjective measure complements objective neurological scores to assess the restoration of volitional control. Research using AMA or similar scales focuses on the neurophysiological evaluation of residual motor function and recovery patterns below the injury level [30] and related functional outcome research in SCI [31].

### Sample Size Justification

An a priori power analysis was conducted (G*Power version 3.1) to determine the necessary sample size for a one-way ANCOVA with three groups and one covariate. To detect a large effect size (f=0.45) based on previous neurostimulation literature, with a significance level (α) of 0.05 and a statistical power of 80%, a total minimum sample size of 48 participants was calculated. To account for potential dropouts or non-compliance, a total of 50 participants were recruited and randomized.

### Statistical analysis

All statistical analyses were planned and performed according to the intention-to-treat (ITT) principle. There was no sample loss or dropouts, as all 50 randomized participants completed the study and were included in the final analysis. Statistical analyses were performed using Python statistical libraries (SciPy Stats 1.10; Statsmodels 0.14). The normality of data distribution was assessed using the Shapiro-Wilk test, and Levene’s test was used to verify the assumption of homogeneity of variances. Baseline demographic and clinical characteristics were compared across the three groups using One-Way Analysis of Variance (ANOVA) for continuous variables and the Kruskal-Wallis H test for ordinal variables to ensure randomization resulted in balanced groups.

To rigorously evaluate the impact of the interventions on continuous primary outcomes (ASIA Lower Extremity Motor Scores and Light Touch Sensory Scores), a One-Way Analysis of Covariance (ANCOVA) was conducted. The post-intervention score was defined as the dependent variable, the Treatment Group (Reference, NEUROM, NEUROM+tDCS) as the fixed factor, and the baseline (pre-intervention) score as the covariate.

For ordinal outcome measures (Assessment of Movement Attempt [AMA] Intensity and Frequency), non-parametric tests were employed. Within-group changes (Pre vs. Post) were analyzed using the Wilcoxon Signed-Rank Test. Between-group differences at the post-intervention time point were analyzed using the Kruskal-Wallis H Test. Where significant between-group differences were found, Dunn’s post-hoc tests were performed with Bonferroni adjustment to identify specific group differences.

Data are presented as mean (SD) for normally distributed continuous variables and as median (25th–75th percentiles) for ordinal or non-normally distributed data, adhering to the journal’s formatting guidelines. All reported p-values are two-tailed, with statistical significance set at.

## Results

### Baseline characteristics and chronicity

Demographic and clinical characteristics for all 50 randomized participants are detailed in Table 1. There were no statistically significant differences between the three groups regarding age (p=0.57), gender (p=0.92), neurological level of injury (p=0.96), or baseline ASIA Impairment Scale (AIS) grade (p=0.75). Crucially, the study population represented a highly chronic cohort, with a mean time since injury of 15.4 ± 3.16 months (Range: 9–22 months). This extended duration—well beyond the 12-month window where spontaneous neurological recovery typically plateaus—confirms that the functional improvements observed in this trial are attributable to the therapeutic interventions rather than natural history. Furthermore, the distribution of injury severity was balanced, with 30% of participants classified as AIS A (motor-complete) and 70% as AIS B/C (incomplete), ensuring that injury completeness did not confound the treatment effects.

**Table 1:** Baseline Demographic and Clinical Characteristics.

Characteristic	Reference (n = 10)	NEUROM (n = 20)	NEUROM+tDCS (n = 20)	P-value
Age (years), Mean (SD)	28.6 (8.0)	29.8 (5.4)	31.1 (6.1)	0.57 {a}
Time since Injury (months), Mean (SD)	13.7 (2.5)	16.1 (3.2)	15.5 (3.2)	0.14 {a}
Gender (Male/Female), n	8/2	16/4	15/5	0.92 {b}
Neurological Level, n (%)				0.96 {b}
Thoracic (T1–T10)	4 (40%)	7 (35%)	7 (35%)	–
Thoracolumbar (T11–L1)	6 (60%)	13 (65%)	13 (65%)	–
ASIA Impairment Scale (AIS), n (%)				0.75 {b}
AIS A (Complete)	3 (30%)	5 (25%)	7 (35%)	–
AIS B (Sensory Incomplete)	7 (70%)	14 (70%)	11 (55%)	–
AIS C (Motor Incomplete)	0 (0%)	1 (5%)	2 (10%)	–

Note: SD = Standard Deviation. {a} One-way ANOVA; {b} Chi-square test.

### Motor Recovery (LEMS)

**Fig 3:**
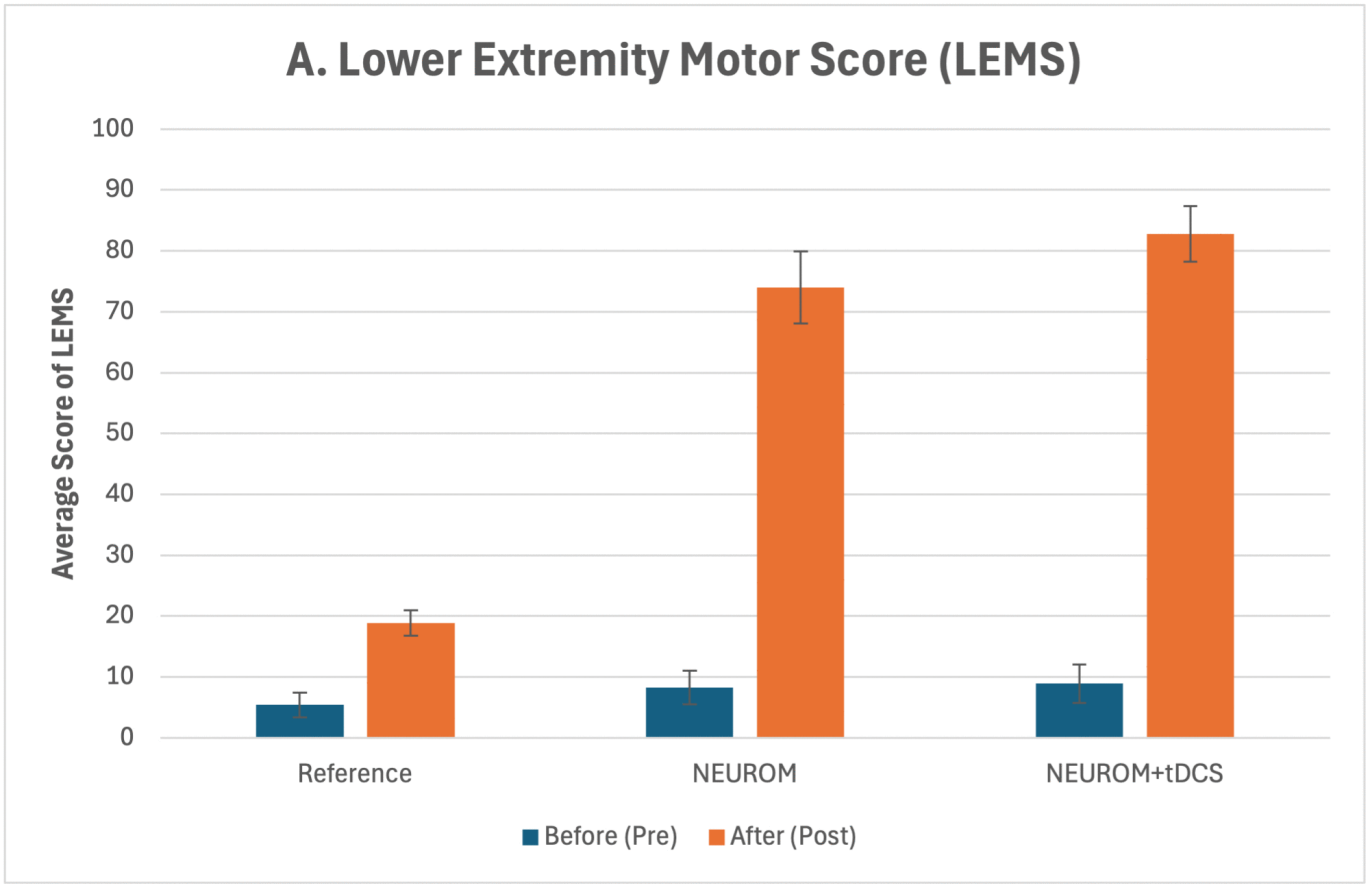
Effects of NEUROM and tDCS interventions on Lower Extremity Motor Score (LEMS) recovery. Mean change in Lower Extremity Motor Score (LEMS) recovery. The NEUROM+tDCS group demonstrated a significantly greater additive improvement compared to both the NEUROM-only (* P < 0.05) and Reference ( P < 0.001) groups, indicating a specific benefit of tDCS on motor output. Note: Total analyzed sample size *N* = 50 (Reference: *n* = 10; NEUROM: *n* = 20; NEUROM+tDCS: *n* = 20). Bar charts display Mean + Standard Deviation (SD). * P < 0.05,  P < 0.001.

The Reference group demonstrated a stable baseline with no significant motor recovery (mean pre-to-post change: 0.2 (0.6) points). To isolate the effect of the intervention, a one-way ANCOVA was conducted on post-intervention Lower Extremity Motor Scores (LEMS) with baseline scores as a covariate. The analysis revealed a highly significant main effect of the treatment group F(2, 46)=53.1, p<0.001, with a large effect size (partial $\overset{\text{2}}{\underset{p}{\eta }}=\text{0}.\text{7}\text{0}$). Post-hoc analysis of the estimated marginal means confirmed that the **NEUROM+tDCS** group achieved the superior outcome (mean pre-to-post increase: 18.8 (4.6) points). This was significantly higher than both the **NEUROM**group (mean increase: 14.3 (5.6) points;) and the Reference group p<0.001. These results indicate that while the NEUROM protocol alone improves motor function, the addition of tDCS provides a statistically significant additive benefit (Fig 3).

### Sensory recovery (light touch and pin prick)

Regarding sensory function, the ANCOVA indicated a significant main effect of group for both modalities, with large effect sizes observed for each.

- **Light Touch:** Both active groups demonstrated better improvements compared to the Reference group 0.3(0.5); $ϝ(\text{2},\;\text{4}\text{6})=\text{2}\text{3}\text{6}.\text{9},\;p<\text{0}.\text{0}\text{0}\text{1});\;\overset{\text{2}}{\underset{p}{\eta }}=\text{0}.\text{9}\text{1}$. The NEUROM group showed a mean change of 58.4 (9.6) points, and the NEUROM+tDCS group showed a mean change of 62.8 (10.3) points.
- **Pin Prick:** Similarly, for pain sensation, significant group differences were found $F(\text{2},\;\text{4}\text{6})=\text{1}\text{7}\text{8}.\text{8},\;p<\text{0}.\text{0}\text{0}\text{1};\;\overset{\text{2}}{\underset{p}{\eta }}=\text{0}.\text{8}\text{9}$. The NEUROM group showed a mean change of 56.1 (10.8) points, while the NEUROM+tDCS group showed a mean change of 60.85 (10.6) points.

**Fig 4:**
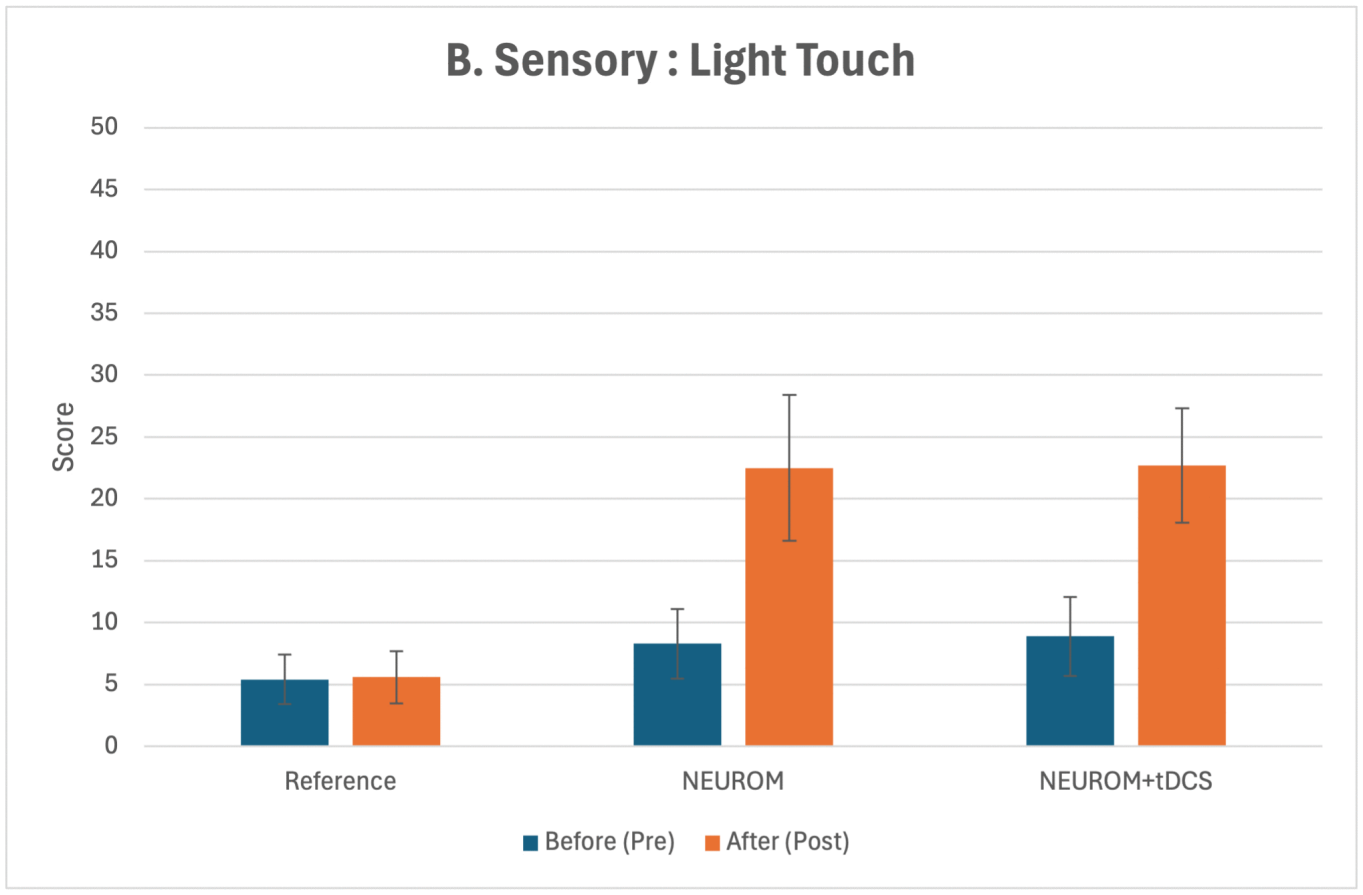
Effects of NEUROM and tDCS interventions on Light Touch sensory recovery. Mean change in sensory recovery for Light Touch scores. Both active groups (NEUROM and NEUROM+tDCS) improved significantly compared to the Reference group ( P < 0.001) across the sensory modality, with no statistically significant difference observed between the two active intervention arms.

**Fig 5:**
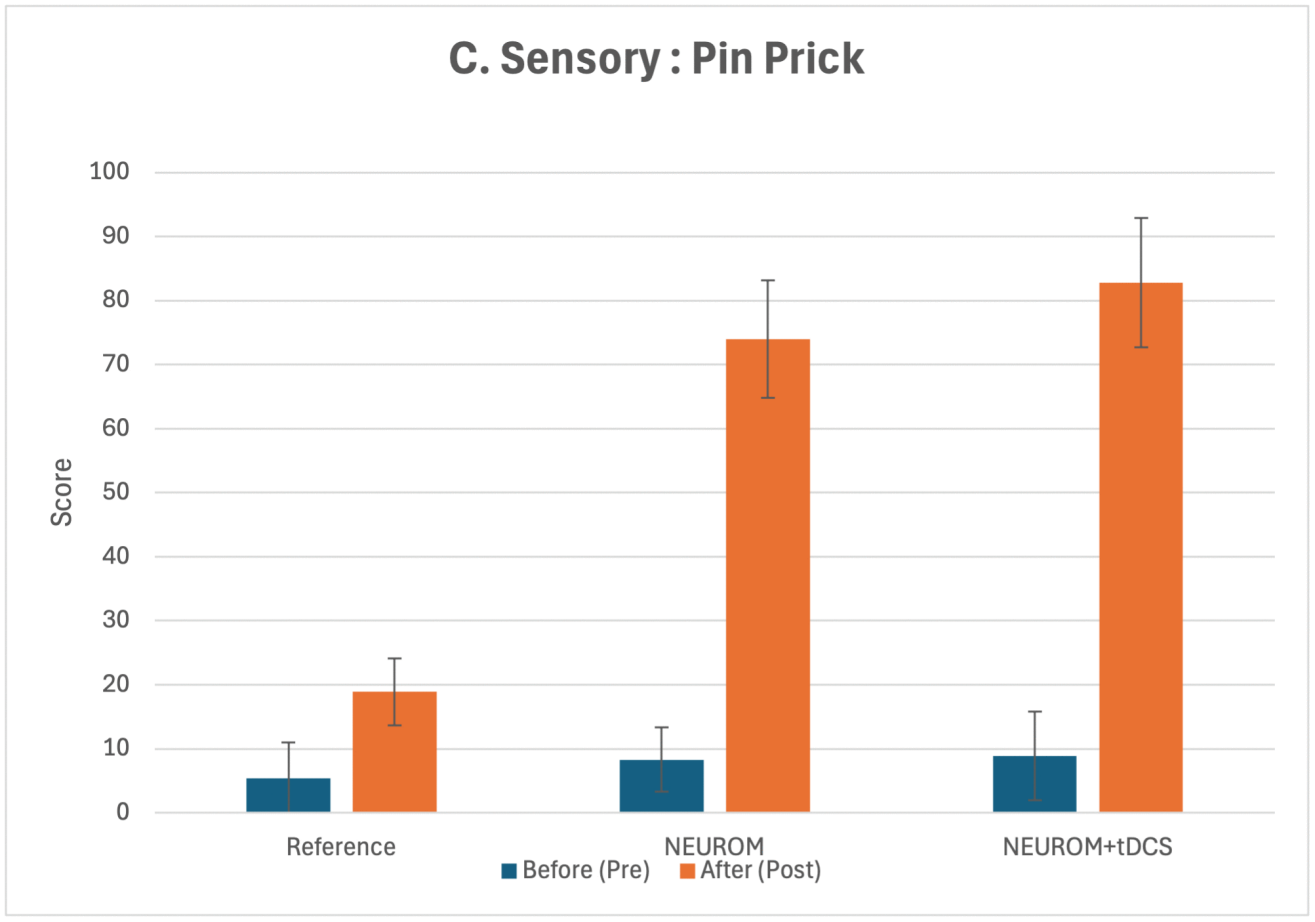
Effects of NEUROM and tDCS interventions on Pin Prick sensory recovery. **Mean change in sensory recovery for Pin Prick scores.** Both active groups (NEUROM and NEUROM+tDCS) improved significantly compared to the Reference group (*P* < 0.001) across the sensory modality, with no statistically significant difference observed between the two active intervention arms.

Crucially, for both Light Touch and Pin Prick, the difference between the NEUROM and NEUROM+tDCS groups was not statistically **significant** p>0.05. This consistent finding across both sensory modalities suggests that the peripheral recruitment component of the NEUROM protocol was the primary driver of sensory recovery, with tDCS providing no significant additional gain in this domain (Fig 4 and 5).

### Volitional Drive (AMA) Intensity of Attempt

**Fig 6:**
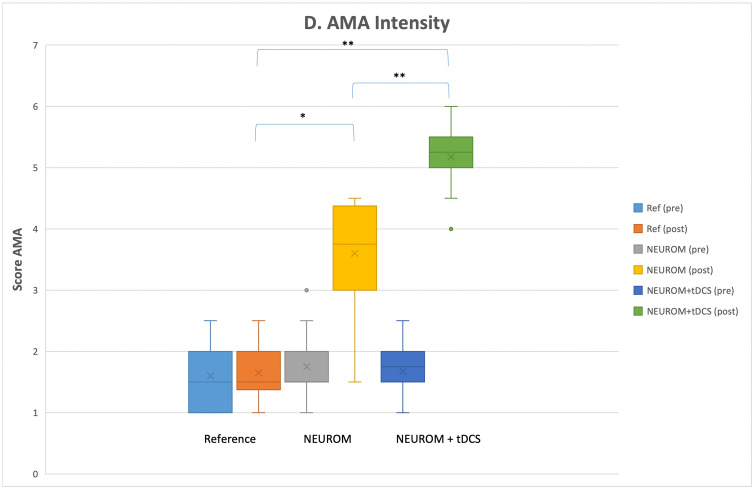
Subjective Assessment of Movement Attempt (AMA) Intensity scores. Evaluation of the Intensity of subjective movement attempts. The combined NEUROM+tDCS group reported significantly higher intensity of limb use compared to both the NEUROM and Reference groups ( P < 0.001), aligning with the objective motor gains.

A Kruskal-Wallis test showed significant differences in the subjective intensity of motor attempts between groups (H=39.89, p<0.001)). The NEUROM+tDCS group reported the highest intensity [Median: 5.2 (25th–75th percentiles: 5.0–5.5)], representing a “strong/vivid” feeling of movement. This was significantly higher than the Reference group [Median: 1.5 (1.5–2.0); p < 0.001] and the NEUROM group [Median: 3.8 (3.0–4.1); p < 0.001] (Fig 6).

**Fig 7:**
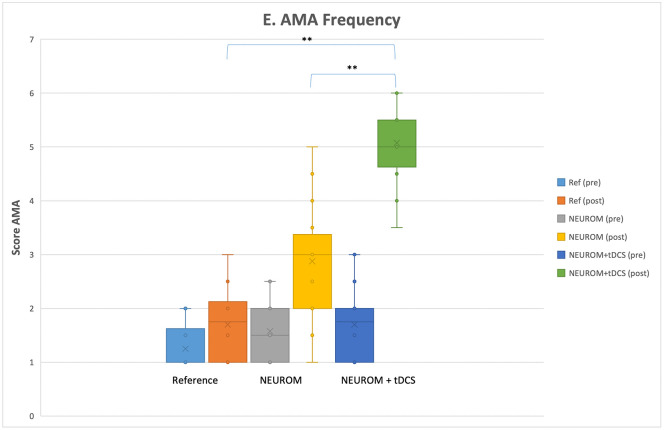
Subjective Assessment of Movement Attempt (AMA) Frequency scores. **Evaluation of the Frequency of subjective movement attempts.** The combined NEUROM+tDCS group reported significantly higher frequency of limb use compared to both the NEUROM and Reference groups ( P < 0.001), aligning with the objective motor gains.

*Frequency of Spontaneous Use:* Similarly, the frequency of spontaneous limb use in daily life differed significantly across groups (p < 0.001). The combined group reported the highest frequency [Median: 5.0 (4.9–5.5)], significantly exceeding the NEUROM group [Median: 3.0 (2.0–3.1); p < 0.001] and the Reference group [Median: 1.8 (1.0−2.0);p<0.001] (Fig 7).

## Discussion

### The combined effect of central and peripheral activation

Despite the limited capacity for spontaneous regeneration in the adult CNS, individuals with incomplete SCI often demonstrate substantial recovery. This recovery is likely due to the reorganization of spared neural circuits and synaptic plasticity [32]. Our findings confirm that significant functional recovery is achievable in chronic SCI through targeted reorganization. Neural plasticity after SCI, including synaptic remodeling, axonal sprouting, and circuit reorganization, is a major contributor to spontaneous and therapy-driven functional recovery. Recent reviews examine enhancing plasticity through spinal interneuron engagement and various therapeutic approaches targeting these mechanisms [33,34]. This study demonstrates a clear hierarchical benefit: while the NEUROM protocol alone outperformed conventional rehabilitation, the combination of NEUROM and tDCS produced better improvements.

For **motor function**, the combination of NEUROM and tDCS produced better improvements, significantly outperforming both the Reference group and the NEUROM-only group. This confirms an additive effect where central modulation (tDCS) amplifies the benefits of peripheral training. Interestingly, for **sensory function** (Light Touch and Pin Prick), both active groups achieved massive gains compared to the Reference group, but the addition of tDCS did not yield a statistically significant advantage over NEUROM alone. This suggests that the intense peripheral stretching and sensory recruitment inherent to the NEUROM protocol may be sufficient to maximally drive sensory plasticity, potentially reaching a “ceiling effect” that tDCS over the *motor* cortex does not further enhance.

### Mechanism of recovery: Closing the sensorimotor loop

The superior performance of the NEUROM + tDCS group supports the hypothesis of a bidirectional enhancement of plasticity. We propose that tDCS “primes” the primary motor cortex (M1), lowering the activation threshold for motor neurons [32,35]. Simultaneously, the NEUROM protocol provides intense bottom-up peripheral sensory input (via stretching and vibration) while engaging top-down motor planning (via imagery). By synchronizing the “intent to move” (facilitated by tDCS and imagery) with “sensory feedback” (provided by NEUROM), we probably facilitated Hebbian plasticity—strengthening the synaptic connections in spared interneuron networks [36]. This aligns with recent literature suggesting that combining central modulation with peripheral feedback is far more effective than either modality in isolation [37,16].

### Restoration of volitional drive (AMA assessment)

A critical behavioral and neural correlate of recovery is the patient’s ability to generate a “movement attempt.” Our analysis of the AMA scores showed that patients in the combined intervention group reported significantly higher intensity of movement attempts (Median 5.0 vs. 1.5 in the reference group), reflecting a robust restoration of volitional motor drive rather than merely a reduction in effort. This aligns with findings in recent literature where combining tDCS with motor imagery enhances the excitability of motor-related cortical areas, thus strengthening the neural signals associated with movement attempts [38].

The tDCS-induced increase in cortical excitability likely made the motor imagery tasks more vivid and helped patients experience a clearer, more direct neural connection to their paralyzed limbs. This neurophysiological facilitation has been reported to amplify the sense of agency and promote recovery of motor control in clinical populations (e.g., stroke and spinal cord injury patients) through enhanced motor relearning processes [39].

Importantly, this enhancement of volitional drive was not limited to clinical assessments but translated into everyday behavior. Patients in the combined group reported significantly higher frequency of spontaneous limb use, indicative of increased confidence and integration of regained motor control into daily activities. Such transfer of gains from structured rehabilitation to spontaneous functional use is crucial and has been documented in related studies evaluating the functional benefits of tDCS combined with mental imagery or robotic rehabilitation methods [40].

While the results of this study are promising, they must be interpreted within the context of several limitations. First, the intervention period was relatively short (3 weeks), and the sample sizes were modest with an uneven distribution; while the smaller reference group (n = 10) was designed primarily to rule out spontaneous recovery, this inequality remains a statistical limitation. Second, due to the active nature of the physical interventions, it was not possible to double-blind the participants or treating therapists, though outcome assessors remained strictly blinded. Finally, the current findings are based solely on a specific sample derived from our local population.

## Conclusion

This trial suggests that combining central excitability (via tDCS) with intense peripheral neuromotor recruitment (the NEUROM protocol) may offer a clinically valuable strategy for enhancing sensorimotor recovery in chronic spinal cord injury. Rather than replacing compensatory rehabilitation, our data indicate that synchronizing central and peripheral interventions could serve as a beneficial adjunct to maximize functional gains. However, future randomized controlled trials with much larger sample sizes, conducted across diverse geographical areas, and including longitudinal follow-ups are essential to confirm the long-term effectiveness and clinical sustainability of the NEUROM+tDCS protocol.

## Supporting information

S1 File CONSORT 2025 Checklist. The completed CONSORT institutional checklist detailing compliance with reporting standards for randomized controlled trials.
(DOCX)

S2 File Request for Ethical Opinion Document. The official documentation and ethical clearance application submitted to the institutional review framework, presented without institutional logos.
(PDF)

S3 File Detailed Research Protocol Document. The comprehensive study protocol outlining the specific methodologies, interventions, and safety monitoring plans for the clinical trial evaluating the NEUROM and tDCS interventions.
(PDF)

S1 Fig Abstract figure. (PNG)
