# Supplementary material for: Additive effect of tDCS and neuromotor recruitment on functional recovery in chronic paraplegia: A randomized controlled trial
Source: PLoS One. 2026 Jun 23;21(6):e0352320. doi: 10.1371/journal.pone.0352320 (PMC13289888; doi:10.1371/journal.pone.0352320)
Supplement: S2 File — The official documentation and ethical clearance application submitted to the institutional review framework, presented without institutional logos. (PDF) [file pone.0352320.s002.pdf]

REQUEST FOR AN ETHICAL OPINION ON A RESEARCH PROTOCOL

Researchers’ name: Ahmad Rifai Sarraj, Ahmad Diab

University title: Professor, Assistant Professor

Faculty: Lebanese University, Faculty of Public Health

Specialty: Physical Therapy

Telephone: 03 662956

Email: ahmadrifaisarraj@ul.edu.lb

ahmaddiab_87@hotmail.com

1. Project information:

1.1 Topic: Neurorehabilitation

1.2 Place: Rahma Hospital for Rehabilitation

1.3 Duration: 2 years starting from May 2019

1.4 Date of beginning of the study: May 2019

1.5 Objectives:

Primary Objective: To evaluate the effectiveness of the NEUROM protocol (with and without tDCS) on lower limb motor function (LEMS) and sensory function (Light Touch/Pin Prick) in chronic paraplegic patients.

Secondary Objective: To assess changes in the volitional intent to move, as measured by the Assessment of Movement Attempt (AMA) scale.

1.6 Type of study: Randomized single-blinded controlled clinical trial

1.7 Research team:

	Name	Position	email
Principal Investigator	Ahmad Rifai Sarraj	Professor	0096103662956 – ahmadrifaisarraj@gmail.com
Co-investigator	Ahmad Diab	Assistant Professor	009613143647 – ahmaddiab_87@hotmail.com
Student	Pamela Nohra	Master’s Student	0096170567849 – nohrapamela@hotmail.com

1.8 Financial resources:

Lebanese University.

1.9 Conflict of interest:

None

1.10 Was this protocol submitted to the ethical committee before?

No

2. Technical information:

2.1 Project involving people:

Describe the participating population (ethnic context, sex, health status, age…):

Target Population

The study will recruit individuals with chronic traumatic spinal cord injury (TSCI) resulting in paraplegia. Participants will be drawn from the patient populations of Rahma Hospital for Rehabilitation (Tripoli, North Lebanon) and the Physical Therapy Center of the Faculty of Public Health (Lebanese University, Beirut).

Demographic Characteristics

- Ethnic Context: The population consists primarily of Lebanese nationals.

from diverse socioeconomic backgrounds, reflecting the patient demographic of the participating public and private rehabilitation centers.

- Age: Adults and adolescents between the ages of 16 and 45 years. This age range is selected to minimize the confounding effects of pediatric growth or geriatric comorbidities.
- Sex: Both male and female participants will be recruited. Based on local epidemiology of spinal cord injury, a higher prevalence of male participants is anticipated (approximate ratio 3:1), but no sex-based exclusion will be applied.

Health Status and Clinical Profile

- Diagnosis: Participants must have a confirmed diagnosis of traumatic spinal cord injury.
- Chronicity: All participants will be in the chronic phase of recovery, defined as at least 3 months post-injury, with a stable neurological status (no fluctuation in AIS score for >1 month).
- Neurological Level: The injury level must be lower dorsal (T1–T10) or thoracolumbar (T11–L1), sparing upper limb function to allow for independent transfer and handling of the tDCS/NEUROM equipment if necessary.
- Severity: The study includes a broad spectrum of injury severities, ranging from Complete (AIS A) to Sensory/Motor Incomplete (AIS B and C), provided they meet the specific safety criteria for tDCS.
- Exclusions: Patients with severe spasticity (Modified Ashworth Scale 3), traumatic brain injury, neuropsychiatric disorders, or medical instability will be excluded to ensure safety and protocol compliance.

Vulnerable population: No

Informed consent required: Yes

Recruitment: Monthly list provided by Rahma Hospital and faculty of public health (Physiotherapy Center) staff; access under staff supervision.

2.2 Risks and benefits:

Potential risks: None (tDCS and other techniques considered safe)

Compensation: Covered by Rehabilitation Hospital Insurance and Healthcare System

Advantages: Accelerated recovery after traumatic spinal injury

Payment: No

Other protocol participation: No

Placebo used: No

2.3 Outcome measures:

Assessments will be performed at **Baseline** (Pre-intervention) and at **Week 3** (Post-intervention).

Primary Outcomes

International Standards for Neurological Classification of SCI (ISNCSCI/ASIA):
* **Lower Extremity Motor Score (LEMS):** Sum of 5 key muscles per leg (0-5 scale). Max score: 50.
* **Sensory Scores:** Light Touch and Pin Prick assessed at 28 key dermatomes. Max score: 112 (Lower limb specific sub-scores will be analyzed).

Secondary Outcome

Assessment of Movement Attempt (AMA):

- A structured interview quantifying the subjective "will" to move.
- **Intensity:** 1 (very weak) to 6 (very high).
- **Frequency:** 1 (rare) to 6 (often).

Certification:

We certify having accepted the scientific and ethical responsibility of the study. Research will not begin before Research Ethics Board (REB) authorization. Any changes or adverse effects will be reported immediately.

Names:

Ahmad Rifai Sarraj

Ahmad Diab
