# Supplementary material for: Additive effect of tDCS and neuromotor recruitment on functional recovery in chronic paraplegia: A randomized controlled trial
Source: PLoS One. 2026 Jun 23;21(6):e0352320. doi: 10.1371/journal.pone.0352320 (PMC13289888; doi:10.1371/journal.pone.0352320)
Supplement: S3 File — The comprehensive study protocol outlining the specific methodologies, interventions, and safety monitoring plans for the clinical trial evaluating the NEUROM and tDCS interventions. (PDF) [file pone.0352320.s003.pdf]

LEBANESE UNIVERSITY
FACULTY OF PUBLIC HEALTH
Centre Azm for Research in Biotechnology and its Applications

REQUEST FOR ETHICAL OPINION ON A RESEARCH PROTOCOL

Date of Submission: January 15, 2019
Protocol Version: 1.0

1. PROJECT INFORMATION

1.1. Project Title:
Effectiveness of a New Neural Motor Recruitment Method (NEUROM) Combined with Transcranial Direct Current Stimulation (tDCS) to Induce and Guide Motor Recovery in Paraplegic Patients.

1.2. Researchers:

- **Principal Investigator:** Prof. Ahmad Rifai Sarraj
  - *Title:* Professor, Department of Physical Therapy
  - *Affiliation:* Faculty of Public Health, Lebanese University
  - *Email:* ahmadrifaisarraj@ul.edu.lb | *Phone:* 03/662956
- **Co-Investigator:** Ahmad Diab
  - *Title:* Assistant Professor
  - *Email:* ahmaddiab_87@hotmail.com

1.3. Study Setting:

- Rahma Hospital for Rehabilitation (Tripoli, North Lebanon)
- Physical Therapy Center, Faculty of Public Health (Hadath, Beirut)

1.4. Proposed Duration:
2 years (Anticipated Start Date: December 2022)

1.5. Objectives:
In this study, we propose a new experimental rehabilitative protocol for Traumatic Spinal Cord Injury (TSCI) patients called the **Neural Motor Recruitment Method (NEUROM)**. This method is based on histological and functional reorganization models following TSCI, Motor Imagery (MI) concepts, and targeted sensory inputs related to motor recovery. We hypothesize that this new method can enhance sparing-induced plasticity and increase motor and sensory recovery in SCI patients, especially when

combined with Transcranial Direct Current Stimulation (tDCS).

2. SUMMARY AND RATIONALE

Background:
Traumatic Spinal Cord Injury (TSCI) is a catastrophic event resulting in motor paralysis and sensory loss. While spontaneous recovery is possible in the acute phase, chronic paraplegia is traditionally viewed as a stable condition with limited potential for further recovery. Recent evidence suggests that the adult spinal cord retains significant plasticity even in the chronic phase. We propose a "bottom-up" approach using a specific motor recruitment protocol (NEUROM) combined with a "top-down" cortical prime using tDCS to enhance this plasticity.
Hypothesis:
We hypothesize that the NEUROM method, especially when combined with anodal tDCS, will induce greater sensorimotor recovery than conventional therapy alone in patients with chronic paraplegia.

3. METHODOLOGY

3.1. Study Design:
Single-blind, randomized controlled clinical trial.

3.2. Participants:

- **Target Sample Size:** $N = 50$
- **Inclusion Criteria:**
    1. Age between 16 and 45 years.
    2. Diagnosis of traumatic spinal cord injury (TSCI).
    3. Chronic phase (3 months post-injury).
    4. Level of injury: Lower dorsal (T1–T10) or Thoracolumbar (T11–L1).
    5. ASIA Impairment Scale (AIS) grade A, B, or C.
- **Exclusion Criteria:**
    1. Traumatic Brain Injury (TBI) or neuropsychiatric comorbidities.
    2. Severe spasticity (Modified Ashworth Scale).
    3. Contraindications to tDCS (metal in head, pacemaker, history of seizures).
    4. Use of sodium channel blockers (e.g., carbamazepine).

3.3. Randomization:
Participants will be randomly assigned into three groups using a web-based random number generator (www.random.org) in a 1:2:2 ratio:

1. **Reference Group ($n = 10$):** Conventional physical therapy.
2. **NEUROM Group ($n = 20$):** Neural Motor Recruitment protocol.
3. **NEUROM + tDCS Group ($n = 20$):** NEUROM protocol + tDCS.

4. GENERAL EXPERIMENTAL PROCEDURE

4.1. Conventional Rehabilitation Program (Reference Group)
Patients will receive a standardized conventional program including:

- Passive and active mobilization.
- Strengthening and endurance exercises.
- Neuromuscular facilitation techniques.
- **Dosage:** 5 sessions/week for 3 weeks.

4.2. Neural Motor Recruitment (NEUROM) Method
This closed-loop protocol consists of two phases:

- **Phase 1 (Week 1):** Daily Motor Imagery (MI) training combined with 20 minutes of static stretching (hamstrings/soleus).
- **Phase 2 (Weeks 2-3):** Active NEUROM sessions.
  - **Visual:** Patients view a 5-minute video of feet performing dorsi- and plantarflexion on a large screen (43-inch, 3.5m distance).
  - **Cognitive:** Audio instructions guide the patient to perform Kinesthetic Motor Imagery ("Imagine yourself performing the movement...").
  - **Sensory:** Concurrent peripheral sensory stimulation.

4.3. Transcranial Direct Current Stimulation (tDCS)

- **Device:** Starstim NE wireless neurostimulator (Neuroelectrics®, Barcelona).
- **Montage:** Bi-hemispheric (C3/C4). Alternating anode/cathode placement on Left/Right M1 daily.
- **Parameters:** 1 mA intensity, 20 minutes duration, 7-second ramp.
- **Safety:** A neurologist will screen patients for contraindications. A specific questionnaire for adverse effects (headache, skin irritation) will be administered before and after each session.

5. OUTCOME MEASURES

Time Points: Baseline (Pre) and Week 3 (Post-Intervention).

1. **ASIA Impairment Scale (ISNCSCI):**
   - **Motor Score (LEMS):** 5 key muscles per leg (0-5 scale). Max = 50.
   - **Sensory Score:** Light Touch and Pin Prick at 12 key points per leg. Max = 48 each.
2. **Assessment of Movement Attempt (AMA):**
   - Structured interview rating the **Intensity** (1-6) and **Frequency** (1-6) of the attempt to move the paralyzed limb.

6. CONFIDENTIALITY AND DATA SECURITY

6.1. Data Protection:

- All participant data will be coded to ensure anonymity.
- Paper records will be stored in locked cabinets at the Faculty of Public Health.
- Digital data will be stored on password-protected computers accessible only to the research team.

6.2. Access to Data:

- Only the Principal Investigator (Prof. Ahmad Rifai Sarraj) and authorized co-investigators will have access to the raw data.
- Data will be retained for 3 years post-study for verification purposes and then destroyed.

7. ETHICAL CONSIDERATIONS

- **Informed Consent:** Written informed consent will be obtained from all participants in Arabic (their native language). The consent form explains the study purpose, procedures, risks, and the right to withdraw at any time. (See attached *Consent Form*).
- **Safety Monitoring:** A senior research specialist will monitor all sessions. Any adverse events (e.g., skin redness, dizziness) will be recorded and reported immediately. If a patient reports severe symptoms (score 5/10 on the safety questionnaire), they will be withdrawn from the study.
- **Risk/Benefit Analysis:** The risks of tDCS (mild skin irritation, headache) are minimal and transient. The potential benefits (motor recovery) are significant for this patient population.

8. SIGNATURES

I, the undersigned **Ahmad Rifai Sarraj**, certify that I accept the scientific and ethical responsibility for this study. I will not start research before receiving the Ethics Committee's authorization. I agree to respect the guidelines of the Research Ethics Board and to inform them of any changes or adverse events.

Principal Investigator:
Prof. Ahmad Rifai Sarraj
Date: 15 January 2019
