# Supplementary figures and images for: Additive effect of tDCS and neuromotor recruitment on functional recovery in chronic paraplegia: A randomized controlled trial
Source: PLoS One. 2026 Jun 23;21(6):e0352320. doi: 10.1371/journal.pone.0352320 (PMC13289888; doi:10.1371/journal.pone.0352320)

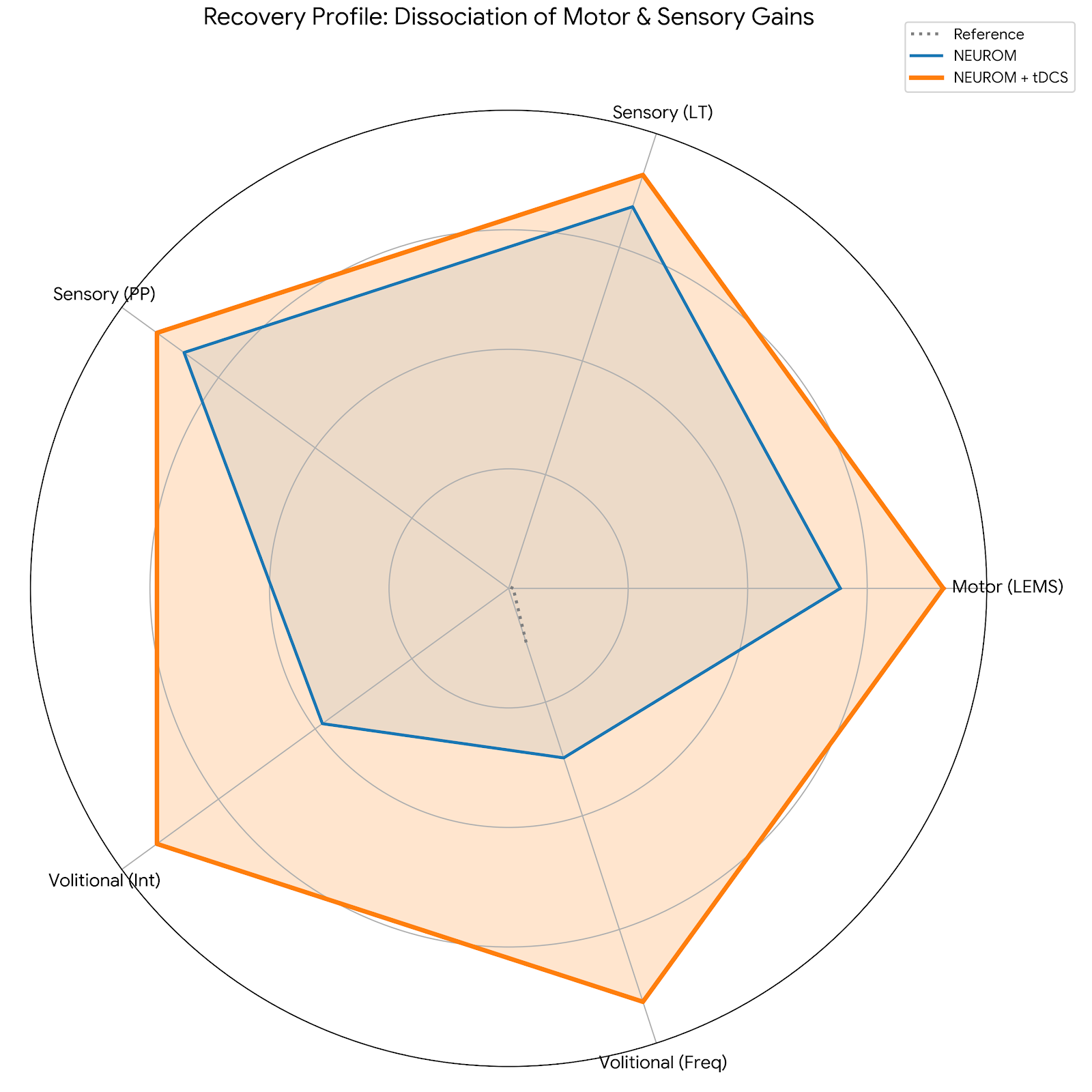

Supplement: S1 Fig — (PNG) [file pone.0352320.s004.png]
