# Supplementary material for: Additive effect of tDCS and neuromotor recruitment on functional recovery in chronic paraplegia: A randomized controlled trial
Source: PLoS One. 2026 Jun 23;21(6):e0352320. doi: 10.1371/journal.pone.0352320 (PMC13289888; doi:10.1371/journal.pone.0352320)
Supplement: Attachment — Submitted filename: Response to Reviewers.docx [file pone.0352320.s006.docx]

**Date:** April 22, 2026

**To:** Dr. Mohammad Sidiq, PhD Physiotherapy, FAIMER Fellow

Academic Editor, *PLOS ONE*

**Re:** Resubmission of Manuscript PONE-D-26-07973

**Title:** Synergistic Effect of tDCS and Neuromotor Recruitment on Functional Recovery in Chronic Paraplegia: A Randomized Controlled Trial

Dear Dr. Sidiq and Reviewers,

We sincerely thank the Academic Editor and the Reviewers for their thorough, constructive, and insightful feedback. We deeply appreciate the recognition of the clinical relevance of our work. Your rigorous review has significantly strengthened the transparency, methodology, and statistical reporting of this manuscript.

We have carefully addressed all comments and revised the manuscript accordingly. Below is our point-by-point response to the issues raised.

**Responses to Journal Requirements and Academic Editor**

**1. Trial Registration Delay & Protocol Changes**

- **Editor Comment:** The manuscript mentions the trial registration... but it appears to be post hoc to the start of the study. The Method section must explain the delay and confirm no protocol changes.
- **Author Response:** We have added a dedicated section titled **"Trial Registration and Ethical Approval Timeline"** to the Methods. We explicitly clarify that the administrative registration was delayed due to a strict, institution-mandated freeze on patient recruitment during the COVID-19 pandemic. We also included the required statement confirming that all ongoing and related trials are registered, and explicitly verified that no modifications were made to the study design after registration.

**2. Blinding Inconsistency**

- **Editor Comment:** It is referred to as "single-blind" (Abstract) and "double-blind" (Methods). Inconsistency means that something went wrong here; please clarify who was blinded.
- **Author Response:** We apologize for this confusing oversight. The study was a single-blind trial with strictly blinded outcome assessment. We have corrected the word "double-blind" in the Methods section. Furthermore, we have added a dedicated **"Blinding"** sub-heading in the Methods detailing that while it was impossible to blind participants or treating therapists due to the active nature of the physical interventions, all Outcome Assessors and Data Analysts were completely blinded to group allocation.

**3. Data Availability Statement**

- **Editor Comment:** The existing statement (available upon request) is against journal policy.
- **Author Response:** We acknowledge this policy. We will upload the raw, de-identified dataset as a Supporting Information file alongside this resubmission to ensure full compliance with *PLOS ONE* open-data guidelines.

**4. Sample Size Justification**

- **Editor Comment:** No specifications (effect size, power, or alpha) are provided for the a priori power analysis.
- **Author Response:** We have added a **"Sample Size Justification"** sub-heading at the beginning of the Statistical Analysis section. We detail the parameters used in G*Power 3.1: identifying a large effect size $(f = 0.45)$, $\alpha= 0.05$, and $80\%$ power, which yielded a required minimum of 48 participants, justifying our randomized sample of 50.

**5. Control Group Description & Protocol Standardization**

- **Editor Comment:** The intervention of the "reference group” needs detailed explanation.
- **Author Response:** We have thoroughly expanded the description of the Reference Group under **"Experimental Procedure."** To further ensure total transparency, reproducibility, and standardization of the NEUROM and Reference protocols, we have deposited our complete, step-by-step clinical methodology on **protocols.io** and provided the direct DOI link within the manuscript text.

**6. Statistical Reporting Issues**

- **Editor Comment:** P-values and other statistics are not fully worked out or formatted wrongly.
- **Author Response:** We have reviewed and corrected all statistical reporting. We identified and fixed a typographical error in our ANCOVA degrees of freedom (corrected from 52 to 46, reflecting our N=50 sample size).

**7. Mechanistic Claims Overstated**

- **Editor Comment:** Mentioning Hebbian plasticity or top-down/bottom-up mechanisms are speculative statements. Be less conclusive.
- **Author Response:** We agree. We have carefully revised the **Discussion** and **Conclusion** sections to tone down these mechanistic claims. We now present these mechanisms as evidence-based proposals rather than definitive conclusions, and we have softened our concluding language to reflect that this protocol is a "beneficial adjunct" rather than a definitive cure.

**Responses to Reviewer #1**

- **Comment 1.1:** The description of the Assessment of Movement Attempt (AMA) Scale is entirely in bold. Please correct this.
- **Author Response:** This formatting error has been corrected.
- **Comment 1.2:** Was the treatment protocol designed based on a previous study? If so, it would be helpful to clarify.
- **Author Response:**  We appreciate the opportunity to clarify the theoretical origins of our protocol. The complete treatment protocol is a synthesis of established clinical practices rather than a direct replication of a single previous study. Specifically, the NEUROM protocol was inspired by general, evidence-based procedures widely reported throughout the spinal cord injury (SCI) neurorehabilitation literature.
- **Comment 1.3:** Did the authors plan analyses according to the intention-to-treat principle? Was there no sample loss?
- **Author Response:** Yes. We have now explicitly added a statement at the beginning of the **Statistical Analysis** section confirming that all analyses were planned and performed according to the intention-to-treat (ITT) principle. We also confirmed in the text that there was zero sample loss, as all 50 randomized participants completed the trial and were included in the final analysis.

**Responses to Reviewer #2**

- **Comment 2.1:** Clarify primary vs. secondary outcomes. Also validation of AMA scale.
- **Author Response:** We have added an explicit clarifying sentence under the **"Outcome Measures"** heading, designating the LEMS and ISNCSCI sensory scores as the primary objective outcomes, and the AMA scale as the secondary subjective outcome. In addition, we don’t have validation of AMA scale. Tha’s why we put it in the secondary outcome. It describes only the subjective feelings of the participant about its ability to movement.
- **Comment 2.2:** The control group size (n=10) is considerably smaller than intervention groups (n=20 each), raising concerns. Limitations: Needs more critical appraisal... small sample size, lack of blinding, short intervention duration.
- **Author Response:** We acknowledge these valid concerns. Regarding the unequal group distribution, we designed the trial with a smaller reference group (n=10) because its primary methodological purpose was simply to rule out the possibility of spontaneous recovery in this highly chronic cohort, thereby isolating and confirming the specific effects of the active interventions. However, we fully recognize that this 1:2:2 allocation remains a statistical limitation. Accordingly, we have entirely rewritten the **"Limitations and Conclusion"** section to explicitly highlight this uneven group distribution, alongside the modest overall sample size, the short 3-week intervention duration, and the fact that the physical nature of the therapy prevented double-blinding..
- **Comment 2.3:** tDCS protocol lacks justification (e.g., rationale for 1 mA intensity, bilateral stimulation, duration).
- **Author Response:** We greatly appreciate this point. To address this directly, we have added a concise, referenced neurophysiological rationale for the 1.0 mA intensity (avoiding the unpredictable calcium-dependent plasticity shifts seen at higher doses) and the bilateral montage to the **Experimental Procedure** section of the manuscript. The complete, step-by-step clinical methodology and extended justification are also formally housed in our protocols.io entry, which is now cited in the text..

**Responses to Reviewer #3**

- **Comment 3.1:** There seems to be errors in the reported ANCOVA degrees of freedom; for example, (2, 52) = 35.6, but with a total n = 50 subjects...
- **Author Response:** We are very grateful to the reviewer for reporting this typographical error. The error degrees of freedom have been mathematically corrected throughout the Results section from 52 to **46** (reflecting N=50, 3 groups, and 1 covariate).
- **Comment 3.2:** The study design as analyzed does not formally test synergy in the statistical interaction sense; hence, the claims of synergy should be reduced.
- **Author Response:** We agree with this precise statistical observation. We have reviewed the manuscript to reduce claims of strict statistical "synergy," reframing the outcome as a "significant additive benefit" and toning down the mechanistic conclusions accordingly.
- **Comment 3.3:** Conclusions should state that the current findings are ONLY based on the samples derived from the specific population, and should allude to future studies with much larger sample sizes and collected at other geographical areas.
- **Author Response:** We completely agree with this precise statistical observation. Because our study design did not include a tDCS-only arm to formally test statistical interaction, we have systematically reviewed the manuscript to remove claims of strict statistical "synergy." Specifically, we have updated the **Title** of the manuscript to reflect an "Additive Benefit," and we have meticulously reframed the terminology throughout the **Abstract, Introduction, Results, and Discussion** sections to describe the outcomes as a "combined effect" or "significant additive benefit." We have also toned down the mechanistic conclusions accordingly to accurately reflect this additive nature without overstating the statistical model.

**Complete Minimal Dataset & Open Access Repository**

As requested, we have verified that the uploaded Supporting Information files (CSV format) constitute the complete minimal dataset required to replicate all statistical tests and figures in this manuscript. To further guarantee permanent, open-access availability, we have also deposited this dataset into a dedicated repository, which is now live and accessible via DOI: [10.5281/zenodo.19692431](https://doi.org/10.5281/zenodo.19692431).

**Response regarding Data Availability:** Thank you for the clarification. We would like to share our data exclusively through the **external Zenodo repository**, as recommended.

Please update our Data Availability statement to reflect that the complete minimal dataset underlying this study is permanently hosted and publicly accessible via the following DOI: [10.5281/zenodo.19692431](https://doi.org/10.5281/zenodo.19692431)..

To prevent redundancy and streamline the publication process, I have removed the duplicate CSV files from the Supporting Information inventory in the submission portal.

Sincerely,

**Professor Ahmad Rifai Sarraj**

Director, MOHAR Research Laboratory

Faculty of Public Health, Lebanese University
