# Supplementary material for: Additive effect of tDCS and neuromotor recruitment on functional recovery in chronic paraplegia: A randomized controlled trial
Source: PLoS One. 2026 Jun 23;21(6):e0352320. doi: 10.1371/journal.pone.0352320 (PMC13289888; doi:10.1371/journal.pone.0352320)
Supplement: Peer review history

# Peer review: Decision Letter 0

**DOI:** 10.1371/journal.pone.0352320.r001

**Authors:** Mohammad Sidiq

15 Apr 2026

PONE-D-26-07973

Synergistic Effect of tDCS and Neuromotor Recruitment on Functional Recovery in Chronic Paraplegia: A Randomized Controlled Trial

PLOS One

Dear Dr. Rifai Sarraj,

Thank you for submitting your manuscript to PLOS ONE. After careful consideration, we feel that it has merit but does not fully meet PLOS ONE’s publication criteria as it currently stands. Therefore, we invite you to submit a revised version of the manuscript that addresses the points raised during the review process.

Please submit your revised manuscript by May 30 2026 11:59PM. If you will need more time than this to complete your revisions, please reply to this message or contact the journal office at plosone@plos.org. When you're ready to submit your revision, log on to https://www.editorialmanager.com/pone/ and select the 'Submissions Needing Revision' folder to locate your manuscript file.

Please include the following items when submitting your revised manuscript:

- A letter that responds to each point raised by the academic editor and reviewer(s). You should upload this letter as a separate file labeled 'Response to Reviewers'.
- A marked-up copy of your manuscript that highlights changes made to the original version. You should upload this as a separate file labeled 'Revised Manuscript with Track Changes'.
- An unmarked version of your revised paper without tracked changes. You should upload this as a separate file labeled 'Manuscript'.

If you would like to make changes to your financial disclosure, please include your updated statement in your cover letter. Guidelines for resubmitting your figure files are available below the reviewer comments at the end of this letter.

If applicable, we recommend that you deposit your laboratory protocols in protocols.io to enhance the reproducibility of your results. Protocols.io assigns your protocol its own identifier (DOI) so that it can be cited independently in the future. For instructions see: https://journals.plos.org/plosone/s/submission-guidelines#loc-laboratory-protocols. Additionally, PLOS ONE offers an option for publishing peer-reviewed Lab Protocol articles, which describe protocols hosted on protocols.io. Read more information on sharing protocols at https://plos.org/protocols?utm_medium=editorial-email&utm_source=authorletters&utm_campaign=protocols.

As the corresponding author, your ORCID iD is verified in the submission system and will appear in the published article. PLOS supports the use of ORCID, and we encourage all coauthors to register for an ORCID iD and use it as well. Please encourage your coauthors to verify their ORCID iD within the submission system before final acceptance, as unverified ORCID iDs will not appear in the published article. *Only*  the individual author can complete the verification step; PLOS staff *cannot*  verify ORCID iDs on behalf of authors.

We look forward to receiving your revised manuscript.

Kind regards,

Mohammad Sidiq, PhD Physiotherapy, FAIMER Fellow

Academic Editor

PLOS One

Journal Requirements:

When submitting your revision, we need you to address these additional requirements.

1. Please ensure that your manuscript meets PLOS ONE's style requirements, including those for file naming. The PLOS ONE style templates can be found at

https://journals.plos.org/plosone/s/file?id=wjVg/PLOSOne_formatting_sample_main_body.pdf and

https://journals.plos.org/plosone/s/file?id=ba62/PLOSOne_formatting_sample_title_authors_affiliations.pdf.

2. Please include a complete copy of PLOS’ questionnaire on inclusivity in global research in your revised manuscript. Our policy for research in this area aims to improve transparency in the reporting of research performed outside of researchers’ own country or community. The policy applies to researchers who have travelled to a different country to conduct research, research with Indigenous populations or their lands, and research on cultural artefacts. The questionnaire can also be requested at the journal’s discretion for any other submissions, even if these conditions are not met.  Please find more information on the policy and a link to download a blank copy of the questionnaire here: https://journals.plos.org/plosone/s/best-practices-in-research-reporting. Please upload a completed version of your questionnaire as Supporting Information when you resubmit your manuscript.

3. Thank you for submitting your clinical trial to PLOS ONE and for providing the name of the registry and the registration number. The information in the registry entry suggests that your trial was registered after patient recruitment began. PLOS ONE strongly encourages authors to register all trials before recruiting the first participant in a study.

As per the journal’s editorial policy, please include in the Methods section of your paper:

a) your reasons for your delay in registering this study (after enrolment of participants started);

b) confirmation that all related trials are registered by stating: “The authors confirm that all ongoing and related trials for this drug/intervention are registered”

4, In the online submission form, you indicated that [All relevant data are within the manuscript and its Supporting Information files UPON REQUEST].

All PLOS journals now require all data underlying the findings described in their manuscript to be freely available to other researchers, either 1. In a public repository, 2. Within the manuscript itself, or 3. Uploaded as supplementary information.

This policy applies to all data except where public deposition would breach compliance with the protocol approved by your research ethics board. If your data cannot be made publicly available for ethical or legal reasons (e.g., public availability would compromise patient privacy), please explain your reasons on resubmission and your exemption request will be escalated for approval.

5.Please include captions for your Supporting Information files at the end of your manuscript, and update any in-text citations to match accordingly. Please see our Supporting Information guidelines for more information: http://journals.plos.org/plosone/s/supporting-information.

6. If the reviewer comments include a recommendation to cite specific previously published works, please review and evaluate these publications to determine whether they are relevant and should be cited. There is no requirement to cite these works unless the editor has indicated otherwise.

Additional Editor Comments (if provided):

The article is an interesting and clinically relevant randomized controlled trial that presents the answer to a particular question in the field of neurorehabilitation. Although the topic is quite timely and the results can make a difference, there are some issues connected to clarity of the methodology, transparency of reporting, and following the journal rules. As it stands, the publication needs significant revision before it can be accepted to the standards of scientific rigor in various aspects, including consistency in the study design, statistical presentation, and availability of data. The response to such issues will enhance the validity and interpretability of findings and enhance the overall quality of the manuscript.

Here are my comments apart from the reviewer observations as under

1. Registration and clarification of the study.

The manuscript mentions the trial registration (NCT04790149), but it appears to be post hoc to the start of the study. The Method section must explain the delay (not just the cover letter) and confirm that there were no protocol changes after registration.

2. Blinding Inconsistency

It is referred to as "single-blind" (Abstract) and "double-blind" (Methods). Inconsistency means that something went wrong here; please clarify who was blinded.

3. Data Availability Statement - non-simultaneous.

The existing statement (available upon request) is against journal policy. Authors are required to save data in an open-source repository, or informative restrictions need to be explained.

4. Sample Size Justification

Although it is stated that a priori power analysis was conducted, no specifications (effect size, power, or alpha) are provided. Kindly provide complete parameters and assumptions.

5. Control Group Description

The intervention of the "reference group” is in need of a more detailed explanation. Discuss the reason this is normal care and whether or not intensity/dosage can be equated with intervention arms.

6. Statistical Reporting Issues

P-values and other statistics are not fully worked out or formatted wrongly (e.g., an empty value in Results).

Always report F-values, degrees of freedom, exact p-values, and sizes of effects.

7. Outcome Measures - Validity and Novelty.

The AMA scale should have better validation or citation support, as it does not seem as standardized as ISNCSCI/LEMS.

8. Short Intervention Duration

Chronic SCI has a fairly low intervention duration of 10 days/3 weeks. Clinical relevance should be better justified by the authors, and effects on sustainability should be discussed.

9. Mechanistic Claims Overstated

The mentioning of the so-called Hebbian plasticity or the top-down and bottom-up mechanisms are both speculative statements. Be less conclusive or provide more supporting evidence.

10. Figures and Reporting

Figures (graphical abstract, Figures 1-3) are mentioned but not wholly incorporated and discussed in the descriptive details.

ensure that figures are of journal quality and are labeled.

[Note: HTML markup is below. Please do not edit.]

Reviewers' comments:

Reviewer's Responses to Questions

**Comments to the Author**

1. Is the manuscript technically sound, and do the data support the conclusions?

The manuscript must describe a technically sound piece of scientific research with data that supports the conclusions. Experiments must have been conducted rigorously, with appropriate controls, replication, and sample sizes. The conclusions must be drawn appropriately based on the data presented.

Reviewer #1: Yes

Reviewer #2: Yes

Reviewer #3: No

**********

2. Has the statistical analysis been performed appropriately and rigorously?

Reviewer #1: Yes

Reviewer #2: Yes

Reviewer #3: No

**********

3. Have the authors made all data underlying the findings in their manuscript fully available?

The PLOS Data policy requires authors to make all data underlying the findings described in their manuscript fully available without restriction, with rare exception (please refer to the Data Availability Statement in the manuscript PDF file). The data should be provided as part of the manuscript or its supporting information, or deposited to a public repository. For example, in addition to summary statistics, the data points behind means, medians and variance measures should be available. If there are restrictions on publicly sharing data—e.g. participant privacy or use of data from a third party—those must be specified.

Reviewer #1: Yes

Reviewer #2: Yes

Reviewer #3: Yes

**********

4. Is the manuscript presented in an intelligible fashion and written in standard English?

PLOS ONE does not copyedit accepted manuscripts, so the language in submitted articles must be clear, correct, and unambiguous. Any typographical or grammatical errors should be corrected at revision, so please note any specific errors here.

Reviewer #1: Yes

Reviewer #2: Yes

Reviewer #3: No

**********

5. Review Comments to the Author

Please use the space provided to explain your answers to the questions above. You may also include additional comments for the author, including concerns about dual publication, research ethics, or publication ethics. (Please upload your review as an attachment if it exceeds 20,000 characters)

Reviewer #1: Overview and general recommendation:

Thank you for giving me the opportunity to review the manuscript entitled “Synergistic Effect of tDCS and Neuromotor Recruitment on Functional Recovery in Chronic Paraplegia: A Randomized Controlled Trial”. The findings of this study are expected to contribute meaningfully to the body of knowledge in the field and support the development of more effective treatment strategies for this population. The study is very well described, following CONSORT guidelines. The research proposal is relevant and aligns with the journal’s scope.

Minor Comments

The description of the Assessment of Movement Attempt (AMA) Scale is entirely in bold. Please correct this.

Was the treatment protocol designed based on a previous study? If so, it would be helpful to clarify.

Did the authors plan analyses according to the intention-to-treat principle? Was there no sample loss?

Reviewer #2: The study addresses an important clinical question and contributes to neurorehabilitation literature.

Study Design and Methodology

The manuscript inconsistently reports the study as single-blind (Abstract) and double-blind (Methods), which creates confusion. Clarification is essential.

The control group size (n=10) is considerably smaller than intervention groups (n=20 each), raising concerns about statistical power and group comparability.

Although randomization is described, allocation concealment details remain insufficient, particularly regarding implementation and sequence security.

No sample size calculation or power analysis details are clearly described in the manuscript body, which is a critical requirement for RCTs.

The NEUROM protocol is described in detail; however:

The standardization and reproducibility of sensory stimulation and therapist-dependent techniques are unclear.

There is no mention of treatment fidelity assessment or inter-therapist reliability.

The tDCS protocol lacks justification (e.g., rationale for 1 mA intensity, bilateral stimulation, duration).

The selected outcomes (LEMS, sensory scores, AMA) are appropriate; The AMA scale lacks sufficient validation details and references for clinical applicability. No primary vs. secondary outcome distinction is explicitly stated.

The significance level (alpha) is missing in the statistical section.

Effect sizes and confidence intervals are not consistently reported.

There is no mention of handling missing data, although authors claim no loss to follow-up.

The use of multiple comparisons correction needs clearer justification and reporting.

The authors claim a “synergistic effect”, but: The absence of a tDCS-only group limits the ability to conclude synergy definitively. The conclusion about “top-down vs. bottom-up mechanisms” is interesting but appears overstated and speculative given the data. Clinical significance (not just statistical significance) is insufficiently discussed.

The Data Availability Statement is non-compliant with PLOS guidelines, as “available upon request” contradicts journal policy.

Trial registration is provided; It is unclear whether the trial was prospectively registered.

Abstract

Overly interpretative (mechanistic conclusions should be toned down).

Include confidence intervals and clarify primary outcomes.

Introduction

Provides good background, but:Some references are outdated.

The research gap and novelty could be more explicitly stated.

Methods Minor grammatical and formatting issues present.

Clarify: Inclusion criteria (e.g., chronicity threshold inconsistencies: >3 months vs. mean 15 months).

Intervention duration inconsistency (10 days vs. 3 weeks mentioned).

Results

Should include effect sizes (optional ) and CI.

Figures (e.g., CONSORT diagram) are referenced but not fully presented in the text.

Discussion

Strength: Integrates neuroplasticity concepts well.

Limitations: Needs more critical appraisal of limitations, especially:

Small sample size

Lack of blinding

Short intervention duration

Overinterpretation should be reduced.

References

Some recent (last 5 years) high-impact studies on tDCS and SCI should be added.

Recommendations for Improvement

Clarify study design (single vs. double blind)

Provide detailed sample size calculation

Improve data availability compliance

Include effect sizes and confidence intervals

Add a clear distinction between primary and secondary outcomes

Tone down causal and mechanistic claims

Expand discussion on clinical implications and limitations

Major Revision Required

The manuscript has strong potential but requires substantial revisions in methodology reporting, statistical transparency, and interpretation before it can meet PLOS ONE publication standards.

Reviewer #3: This manuscript presents data analysis from a three-arm randomized clinical trial in 50 individuals with chronic paraplegia, comparing a reference rehabilitation group, a NEUROM group, and a combined NEUROM+tDCS group.

The topic is of importance, and the study was registered as a RCT within clinicaltrials.gov (with a valid NCT number), and was approved by the respective IRB/Ethics Committee. While the study objectives sound interesting, is important, and on target, some shortcomings were observed in regards to abiding by the CONSORT guidelines for conducting and reporting results of high-quality randomized controlled trials.

1. Methods:

Methods reporting need some work. An orderly manner is suggested, following CONSORT guidelines, without repeating information, such as Trial Design, Participant Eligibility Criteria and settings, Interventions, Outcomes, sample size/power considerations, Interim analysis and stopping rules, Randomization (details on random number generation, allocation concealment, implementation), Blinding issues, etc, should be mentioned. The authors are advised to create "separate subsections" for each of the possible topics (whichever necessary), and that way produce a very clear writeup. They are advised to write it carefully, following nice examples in the manuscript below:

https://www.sciencedirect.com/science/article/pii/S0889540619300010

Specific comments:

(a) For instance, the randomization and allocation concealment should be made very clear (they are NOT the same thing); the trial staff recruiting patients should NOT have the randomization list. Randomization should be prepared by the trial statistician, and he/she would not participate in the recruiting.

(b) More details on the randomization is needed; I could find no such details, other than saying that random numbers were generated via a web-based random generator. Was it a block randomization? What block size? Why was block randomization not conducted, since, BR helps to achieve equal sample sizes in the 2 arms.

(c) There seems to be design inconsistency! The abstract describes the study as single-blind, whereas the Methods section describes it as double-blind, and the randomization section later clarifies that it was effectively single-blind with blinded outcome assessment, since participants and therapists could not be blinded. This is confusing.

(d) Sample size: There doesn't seem to be any formal sample size/power statement provided! This is a serious limitation, given that the authors are analyzing data generated from a clinical trial. On the overall, the sample size seems to be small, wrt. a 3-arm superiority trial, with a variety of endpoints.

(e) Statistical Analysis writeup

(e1) The statistical analysis framework is not fully developed. Although ANCOVA (for continuous outcomes), and nonparametric tests (for ordinal outcomes) were used, there were no confidence intervals reported for treatment effects, no estimated adjusted mean differences, etc.

(e2) There seems to be errors in the reported ANCOVA degrees of freedom; for example, (2, 52) = 35.6, but with a total n = 50 subjects, it's not clear how 52 appears in (2, 52).

(e3) The single primary endpoint was not clearly mentioned, although, multiple outcomes were mentioned.

(e4) The study design as analyzed does not formally test synergy in the statistical interaction sense; hence, the claims of synergy should be reduced.

(e5) How missing data was handled is not clear.

(e6) The analysis was intention-to-treat, yet, the CONSORT flow diagram consists of words like "Excluded from Analysis (give reasons)". Not clear.

2. Results & Conclusions:

(a) The authors should check that any statement of significance should be followed by a p-value in the entire Results section. Otherwise, the Results section look OK; it's pretty straightforward.

(b) Conclusions should state that the current findings are ONLY based on the samples derived from the specific population, and should allude to future studies with much larger sample sizes and collected at other geographical areas to confirm the effectiveness of the NEUROM+tDCS group, over the other groups.

**********

6. PLOS authors have the option to publish the peer review history of their article (what does this mean?). If published, this will include your full peer review and any attached files.

If you choose “no”, your identity will remain anonymous but your review may still be made public.

**Do you want your identity to be public for this peer review?**  For information about this choice, including consent withdrawal, please see our Privacy Policy.

Reviewer #1: No

Reviewer #2: No

Reviewer #3: No

**********

[NOTE: If reviewer comments were submitted as an attachment file, they will be attached to this email and accessible via the submission site. Please log into your account, locate the manuscript record, and check for the action link "View Attachments". If this link does not appear, there are no attachment files.]

To ensure your figures meet our technical requirements, please review our figure guidelines: https://journals.plos.org/plosone/s/figures

You may also use PLOS’s free figure tool, NAAS, to help you prepare publication quality figures: https://journals.plos.org/plosone/s/figures#loc-tools-for-figure-preparation.

NAAS will assess whether your figures meet our technical requirements by comparing each figure against our figure specifications.

# Author comment: Author response to Decision Letter 1

**DOI:** 10.1371/journal.pone.0352320.r002

6 May 2026

Date: April 22, 2026

To: Dr. Mohammad Sidiq, PhD Physiotherapy, FAIMER Fellow

Academic Editor, PLOS ONE

Re: Resubmission of Manuscript PONE-D-26-07973

Title: Synergistic Effect of tDCS and Neuromotor Recruitment on Functional Recovery in Chronic Paraplegia: A Randomized Controlled Trial

Dear Dr. Sidiq and Reviewers,

We sincerely thank the Academic Editor and the Reviewers for their thorough, constructive, and insightful feedback. We deeply appreciate the recognition of the clinical relevance of our work. Your rigorous review has significantly strengthened the transparency, methodology, and statistical reporting of this manuscript.

We have carefully addressed all comments and revised the manuscript accordingly. Below is our point-by-point response to the issues raised.

Responses to Journal Requirements and Academic Editor

1. Trial Registration Delay & Protocol Changes

Editor Comment: The manuscript mentions the trial registration... but it appears to be post hoc to the start of the study. The Method section must explain the delay and confirm no protocol changes.

Author Response: We have added a dedicated section titled "Trial Registration and Ethical Approval Timeline" to the Methods. We explicitly clarify that the administrative registration was delayed due to a strict, institution-mandated freeze on patient recruitment during the COVID-19 pandemic. We also included the required statement confirming that all ongoing and related trials are registered, and explicitly verified that no modifications were made to the study design after registration.

2. Blinding Inconsistency

Editor Comment: It is referred to as "single-blind" (Abstract) and "double-blind" (Methods). Inconsistency means that something went wrong here; please clarify who was blinded.

Author Response: We apologize for this confusing oversight. The study was a single-blind trial with strictly blinded outcome assessment. We have corrected the word "double-blind" in the Methods section. Furthermore, we have added a dedicated "Blinding" sub-heading in the Methods detailing that while it was impossible to blind participants or treating therapists due to the active nature of the physical interventions, all Outcome Assessors and Data Analysts were completely blinded to group allocation.

3. Data Availability Statement

Editor Comment: The existing statement (available upon request) is against journal policy.

Author Response: We acknowledge this policy. We will upload the raw, de-identified dataset as a Supporting Information file alongside this resubmission to ensure full compliance with PLOS ONE open-data guidelines.

4. Sample Size Justification

Editor Comment: No specifications (effect size, power, or alpha) are provided for the a priori power analysis.

Author Response: We have added a "Sample Size Justification" sub-heading at the beginning of the Statistical Analysis section. We detail the parameters used in G*Power 3.1: identifying a large effect size (f = 0.45), α = 0.05, and 80% power, which yielded a required minimum of 48 participants, justifying our randomized sample of 50.

5. Control Group Description & Protocol Standardization

Editor Comment: The intervention of the "reference group” needs detailed explanation.

Author Response: We have thoroughly expanded the description of the Reference Group under "Experimental Procedure." To further ensure total transparency, reproducibility, and standardization of the NEUROM and Reference protocols, we have deposited our complete, step-by-step clinical methodology on protocols.io and provided the direct DOI link within the manuscript text.

6. Statistical Reporting Issues

Editor Comment: P-values and other statistics are not fully worked out or formatted wrongly.

Author Response: We have reviewed and corrected all statistical reporting. We identified and fixed a typographical error in our ANCOVA degrees of freedom (corrected from 52 to 46, reflecting our N=50 sample size).

7. Mechanistic Claims Overstated

Editor Comment: Mentioning Hebbian plasticity or top-down/bottom-up mechanisms are speculative statements. Be less conclusive.

Author Response: We agree. We have carefully revised the Discussion and Conclusion sections to tone down these mechanistic claims. We now present these mechanisms as evidence-based proposals rather than definitive conclusions, and we have softened our concluding language to reflect that this protocol is a "beneficial adjunct" rather than a definitive cure.

Responses to Reviewer #1

Comment 1.1: The description of the Assessment of Movement Attempt (AMA) Scale is entirely in bold. Please correct this.

Author Response: This formatting error has been corrected.

Comment 1.2: Was the treatment protocol designed based on a previous study? If so, it would be helpful to clarify.

Author Response: We appreciate the opportunity to clarify the theoretical origins of our protocol. The complete treatment protocol is a synthesis of established clinical practices rather than a direct replication of a single previous study. Specifically, the NEUROM protocol was inspired by general, evidence-based procedures widely reported throughout the spinal cord injury (SCI) neurorehabilitation literature.

Comment 1.3: Did the authors plan analyses according to the intention-to-treat principle? Was there no sample loss?

Author Response: Yes. We have now explicitly added a statement at the beginning of the Statistical Analysis section confirming that all analyses were planned and performed according to the intention-to-treat (ITT) principle. We also confirmed in the text that there was zero sample loss, as all 50 randomized participants completed the trial and were included in the final analysis.

Responses to Reviewer #2

Comment 2.1: Clarify primary vs. secondary outcomes. Also validation of AMA scale.

Author Response: We have added an explicit clarifying sentence under the "Outcome Measures" heading, designating the LEMS and ISNCSCI sensory scores as the primary objective outcomes, and the AMA scale as the secondary subjective outcome. In addition, we don’t have validation of AMA scale. Tha’s why we put it in the secondary outcome. It describes only the subjective feelings of the participant about its ability to movement.

Comment 2.2: The control group size (n=10) is considerably smaller than intervention groups (n=20 each), raising concerns. Limitations: Needs more critical appraisal... small sample size, lack of blinding, short intervention duration.

Author Response: We acknowledge these valid concerns. Regarding the unequal group distribution, we designed the trial with a smaller reference group (n=10) because its primary methodological purpose was simply to rule out the possibility of spontaneous recovery in this highly chronic cohort, thereby isolating and confirming the specific effects of the active interventions. However, we fully recognize that this 1:2:2 allocation remains a statistical limitation. Accordingly, we have entirely rewritten the "Limitations and Conclusion" section to explicitly highlight this uneven group distribution, alongside the modest overall sample size, the short 3-week intervention duration, and the fact that the physical nature of the therapy prevented double-blinding..

Comment 2.3: tDCS protocol lacks justification (e.g., rationale for 1 mA intensity, bilateral stimulation, duration).

Author Response: We greatly appreciate this point. To address this directly, we have added a concise, referenced neurophysiological rationale for the 1.0 mA intensity (avoiding the unpredictable calcium-dependent plasticity shifts seen at higher doses) and the bilateral montage to the Experimental Procedure section of the manuscript. The complete, step-by-step clinical methodology and extended justification are also formally housed in our protocols.io entry, which is now cited in the text..

Responses to Reviewer #3

Comment 3.1: There seems to be errors in the reported ANCOVA degrees of freedom; for example, (2, 52) = 35.6, but with a total n = 50 subjects...

Author Response: We are very grateful to the reviewer for reporting this typographical error. The error degrees of freedom have been mathematically corrected throughout the Results section from 52 to 46 (reflecting N=50, 3 groups, and 1 covariate).

Comment 3.2: The study design as analyzed does not formally test synergy in the statistical interaction sense; hence, the claims of synergy should be reduced.

Author Response: We agree with this precise statistical observation. We have reviewed the manuscript to reduce claims of strict statistical "synergy," reframing the outcome as a "significant additive benefit" and toning down the mechanistic conclusions accordingly.

Comment 3.3: Conclusions should state that the current findings are ONLY based on the samples derived from the specific population, and should allude to future studies with much larger sample sizes and collected at other geographical areas.

Author Response: We completely agree with this precise statistical observation. Because our study design did not include a tDCS-only arm to formally test statistical interaction, we have systematically reviewed the manuscript to remove claims of strict statistical "synergy." Specifically, we have updated the Title of the manuscript to reflect an "Additive Benefit," and we have meticulously reframed the terminology throughout the Abstract, Introduction, Results, and Discussion sections to describe the outcomes as a "combined effect" or "significant additive benefit." We have also toned down the mechanistic conclusions accordingly to accurately reflect this additive nature without overstating the statistical model.

Complete Minimal Dataset & Open Access Repository

As requested, we have verified that the uploaded Supporting Information files (CSV format) constitute the complete minimal dataset required to replicate all statistical tests and figures in this manuscript. To further guarantee permanent, open-access availability, we have also deposited this dataset into a dedicated repository, which is now live and accessible via DOI: 10.5281/zenodo.19692431.

Response regarding Data Availability: Thank you for the clarification. We would like to share our data exclusively through the external Zenodo repository, as recommended.

Please update our Data Availability statement to reflect that the complete minimal dataset underlying this study is permanently hosted and publicly accessible via the following DOI: 10.5281/zenodo.19692431..

To prevent redundancy and streamline the publication process, I have removed the duplicate CSV files from the Supporting Information inventory in the submission portal.

Sincerely,

Professor Ahmad Rifai Sarraj

Director, MOHAR Research Laboratory

Faculty of Public Health, Lebanese University

Attachment Submitted filename: Response to Reviewers.docx

# Peer review: Decision Letter 1

**DOI:** 10.1371/journal.pone.0352320.r003

**Authors:** Mohammad Sidiq, Mohammad Sidiq

9 Jun 2026

Additive Effect of tDCS and Neuromotor Recruitment on Functional Recovery in Chronic Paraplegia: A Randomized Controlled Trial

PONE-D-26-07973R1

Dear Sarraj,

We’re pleased to inform you that your manuscript has been judged scientifically suitable for publication and will be formally accepted for publication once it meets all outstanding technical requirements.

Within one week, you’ll receive an e-mail detailing the required amendments. When these have been addressed, you’ll receive a formal acceptance letter, and your manuscript will be scheduled for publication.

Kind regards,

Mohammad Sidiq, PhD, Physiotherapy, FAIMER Fellow

Academic Editor

PLOS One

Additional Editor Comments (optional):

Dear authors, I'm pleased to share that the current revision satisfies the quality and rigor as per PLOS ONE standards, and I'm happy to accept this version based on the positive feedback from the reviewers too.

Reviewers' comments:

Reviewer's Responses to Questions

**Comments to the Author**

1. If the authors have adequately addressed your comments raised in a previous round of review and you feel that this manuscript is now acceptable for publication, you may indicate that here to bypass the “Comments to the Author” section, enter your conflict of interest statement in the “Confidential to Editor” section, and submit your "Accept" recommendation.

Reviewer #2: All comments have been addressed

Reviewer #3: All comments have been addressed

**********

2. Is the manuscript technically sound, and do the data support the conclusions?

The manuscript must describe a technically sound piece of scientific research with data that supports the conclusions. Experiments must have been conducted rigorously, with appropriate controls, replication, and sample sizes. The conclusions must be drawn appropriately based on the data presented.

Reviewer #2: Yes

Reviewer #3: (No Response)

**********

3. Has the statistical analysis been performed appropriately and rigorously?

Reviewer #2: Yes

Reviewer #3: (No Response)

**********

4. Have the authors made all data underlying the findings in their manuscript fully available?

The PLOS Data policy requires authors to make all data underlying the findings described in their manuscript fully available without restriction, with rare exception (please refer to the Data Availability Statement in the manuscript PDF file). The data should be provided as part of the manuscript or its supporting information, or deposited to a public repository. For example, in addition to summary statistics, the data points behind means, medians and variance measures should be available. If there are restrictions on publicly sharing data—e.g. participant privacy or use of data from a third party—those must be specified.

Reviewer #2: Yes

Reviewer #3: (No Response)

**********

5. Is the manuscript presented in an intelligible fashion and written in standard English?

PLOS ONE does not copyedit accepted manuscripts, so the language in submitted articles must be clear, correct, and unambiguous. Any typographical or grammatical errors should be corrected at revision, so please note any specific errors here.

Reviewer #2: Yes

Reviewer #3: (No Response)

**********

6. Review Comments to the Author

Please use the space provided to explain your answers to the questions above. You may also include additional comments for the author, including concerns about dual publication, research ethics, or publication ethics. (Please upload your review as an attachment if it exceeds 20,000 characters)

Reviewer #2: The manuscript can be considered for publication in its current form. Thankyou for giving me the opportunity.

Reviewer #3: (No Response)

**********

7. PLOS authors have the option to publish the peer review history of their article (what does this mean?). If published, this will include your full peer review and any attached files.

If you choose “no”, your identity will remain anonymous but your review may still be made public.

**Do you want your identity to be public for this peer review?**  For information about this choice, including consent withdrawal, please see our Privacy Policy.

Reviewer #2: No

Reviewer #3: No

**********

# Editor report: Acceptance letter

**DOI:** 10.1371/journal.pone.0352320.r004

**Authors:** Mohammad Sidiq, Mohammad Sidiq

PONE-D-26-07973R1

PLOS One

Dear Dr. Rifai Sarraj,

I'm pleased to inform you that your manuscript has been deemed suitable for publication in PLOS One. Congratulations! Your manuscript is now being handed over to our production team.

At this stage, our production department will prepare your paper for publication. This includes ensuring the following:

* All references, tables, and figures are properly cited

* All relevant supporting information is included in the manuscript submission,

* There are no issues that prevent the paper from being properly typeset

You will receive further instructions from the production team, including instructions on how to review your proof when it is ready. Please keep in mind that we are working through a large volume of accepted articles, so please give us a few days to review your paper and let you know the next and final steps.

Lastly, if your institution or institutions have a press office, please let them know about your upcoming paper now to help maximize its impact. If they'll be preparing press materials, please inform our press team within the next 48 hours. Your manuscript will remain under strict press embargo until 2 pm Eastern Time on the date of publication. For more information, please contact onepress@plos.org.

You will receive an invoice from PLOS for your publication fee after your manuscript has reached the completed accept phase. If you receive an email requesting payment before acceptance or for any other service, this may be a phishing scheme. Learn how to identify phishing emails and protect your accounts at https://explore.plos.org/phishing.

If we can help with anything else, please email us at customercare@plos.org.

Thank you for submitting your work to PLOS ONE and supporting open access.

Kind regards,

PLOS ONE Editorial Office Staff

on behalf of

Dr. Mohammad Sidiq

Academic Editor

PLOS One
